# Potential marker subset of blood-circulating cytokines on hematopoietic progenitor-to-Th1 pathway in COVID-19

**DOI:** 10.3389/fmed.2024.1319980

**Published:** 2024-02-27

**Authors:** Yasuo Takashima, Tohru Inaba, Tasuku Matsuyama, Kengo Yoshii, Masami Tanaka, Kazumichi Matsumoto, Kazuki Sudo, Yuichi Tokuda, Natsue Omi, Masakazu Nakano, Takaaki Nakaya, Naohisa Fujita, Chie Sotozono, Teiji Sawa, Kei Tashiro, Bon Ohta

**Affiliations:** ^1^Department of Genomic Medical Sciences, Kyoto Prefectural University of Medicine, Kyoto, Japan; ^2^Department of Infection Control and Laboratory Medicine, Kyoto Prefectural University of Medicine, Kyoto, Japan; ^3^Department of Emergency Medicine, Kyoto Prefectural University of Medicine, Kyoto, Japan; ^4^Department of Mathematics and Statistics in Medical Sciences, Kyoto Prefectural University of Medicine, Kyoto, Japan; ^5^Faculty of Clinical Laboratory, University Hospital Kyoto Prefectural University of Medicine, Kyoto, Japan; ^6^Department of Anesthesiology, Kyoto Prefectural University of Medicine, Kyoto, Japan; ^7^Department of Infectious Diseases, Kyoto Prefectural University of Medicine, Kyoto, Japan; ^8^Kyoto Prefectural Institute of Public Health and Environment, Kyoto, Japan; ^9^Department of Ophthalmology, Kyoto Prefectural University of Medicine, Kyoto, Japan; ^10^University Hospital Kyoto Prefectural University of Medicine, Kyoto, Japan

**Keywords:** COVID-19, cytokine storm, blood-circulating cytokine, coefficient of variation, timelapse monitoring

## Abstract

In this study, we analyzed a relatively large subset of proteins, including 109 kinds of blood-circulating cytokines, and precisely described a cytokine storm in the expression level and the range of fluctuations during hospitalization for COVID-19. Of the proteins analyzed in COVID-19, approximately 70% were detected with Bonferroni-corrected significant differences in comparison with disease severity, clinical outcome, long-term hospitalization, and disease progression and recovery. Specifically, IP-10, sTNF-R1, sTNF-R2, sCD30, sCD163, HGF, SCYB16, IL-16, MIG, SDF-1, and fractalkine were found to be major components of the COVID-19 cytokine storm. Moreover, the 11 cytokines (i.e., SDF-1, SCYB16, sCD30, IL-11, IL-18, IL-8, IFN-γ, TNF-α, sTNF-R2, M-CSF, and I-309) were associated with the infection, mortality, disease progression and recovery, and long-term hospitalization. Increased expression of these cytokines could be explained in sequential pathways from hematopoietic progenitor cell differentiation to Th1-derived hyperinflammation in COVID-19, which might also develop a novel strategy for COVID-19 therapy with recombinant interleukins and anti-chemokine drugs.

## Introduction

Severe acute respiratory syndrome coronavirus 2 (SARS-CoV-2) is a novel β-coronavirus that emerged in China in December 2019, leading to the global pandemic known as coronavirus disease 2019 (COVID-19) (1). Reportedly, severe COVID-19 is characterized by hypoxia with the risk of rapid deterioration that may require intensive care support and, in some cases, can progress to acute respiratory distress syndrome (ARDS), multiple organ failure, and death (1). Precision medical care using biomarkers is currently uncertain due to an inadequate understanding of the pathogenesis and heterogeneity among severe COVID-19 patients (2). Moreover, in some severe COVID-19 patients, a dysregulated hyperinflammatory state can occur, consistent with using a glucocorticoid (e.g., dexamethasone), interleukin (IL)-6 receptor inhibitors (e.g., tocilizumab and sarilumab), and a Janus kinase (JAK) inhibitor (e.g., baricitinib) in the treatment of severe disease (3–6). However, the blood signatures of COVID-19 severity are diverse, including immune suppression, myeloid dysfunction, lymphopenia, interferon-derived immunopathology, T-cell activation and exhaustion, and immune senescence (7–11). On the other hand, urinary levels of fatty acids and docosahexaenoic acid (DHA) are increased by approximately 3-fold in the COVID-19 patients, compared to healthy controls, and furthermore, markedly increased levels of PGE2, TXA2, and PGF2α as metabolites of major proinflammatory lipid mediators are also detected in the urine of the COVID-19 patients (12). While in the human lungs, severe COVID-19 is reportedly characterized by widespread neutrophil and macrophage infiltration and T-cell cytokine production (13). Alveolitis with COVID-19 is also caused by altered redox balance, endothelial damage, and thrombosis (13).

Serum concentrations of proinflammatory cytokines are strongly correlated with disease and clinical outcomes and are increased in patients with severe COVID-19 (14). In such cases, the induced expression of inflammatory cytokines, including IL-6 and tumor necrosis factor (TNF)-α, causes systemic inflammation by dysregulation of immune pathways (15, 16). It has been posited that one of the main causes of such hyperinflammation, as well as the development of serious complications, in patients afflicted with COVID-19, is a delayed or impaired type-I interferon (IFN) response as the first line of antiviral defense (17). In addition to IFNs, serum levels of cytokines have been measured for the discovery of prospective inflammation markers in COVID-19 patients (18–21). Disease severity correlates with several immunological cytokine profiles (18, 19) and various patient-related demographic characteristics, including age, sex, and non-infectious comorbidities (19, 22–25). Of those factors, IFN-γ, IL-6, IL-10, and TNF-α have been proposed for use as predictors of disease severity and pharmacological targets in anti-cytokine therapy (16, 20).

In this study, we performed profiling of the expression and coefficient of variation (CV) of 109 kinds of blood-circulating cytokines in peripheral blood samples obtained from 23 COVID-19 patients. Several cytokine signatures associated with COVID-19 were identified. In addition, the patterns of early-phase and late-phase cytokine expression levels between the patient groups by their severity were investigated. Subsequently, our findings revealed cytokine signatures reflecting variable cytokine storms and their immune pathways, as well as the patient’s severity, the hospitalization period, the clinical outcome, and the specific hallmarks of increasing and decreasing severity. These findings are useful for the diagnosis of COVID-19 and may contribute to the further development of safe and effective therapeutic strategies in patients afflicted with the disease.

## Materials and methods

### Clinical samples

Serum samples were obtained from 23 adult COVID-19 patients (age range: 20–91 years) treated at the University Hospital Kyoto Prefectural University of Medicine during the third and fourth waves of COVID-19 incidence in Japan from November 2020 to June 2021. To measure the serum cytokine levels, we obtained a total of 134 samples from the 23 COVID-19 patients and 26 samples from 13 healthy volunteer control subjects not infected with SARS-CoV-2 viruses. The study protocols were approved by the Institutional Review Board at Kyoto Prefectural University of Medicine (ERB-G-109 and ERB-C-1810). All experiments were performed following the institutional guidelines and in accordance with the tenets outlined in the Declaration of Helsinki, and prior written informed consent was obtained from all study participants.

### Peripheral blood cytokine analysis

Serum samples frozen and stored at −80°C prior to thawing were tested for simultaneous quantification of 109 kinds of blood-circulating cytokines via the use of a Bio-Plex Pro™ Human Cytokine Screening Panel, 48-Plex, a Bio-Plex Pro™ Human Chemokine 40-Plex Panel, a Bio-Plex Pro™ Human Inflammation 37-Plex Panel, a Bio-Plex Pro™ Human Th17 Cytokine 15-Plex Panel, and a Bio-Plex Pro™ TGF-β 3-plex Assay (all from Bio-Rad Laboratories, Inc., Hercules, CA) (Supplementary Table S1). All assays were performed using Bio-Plex^®^ Assay Kits (Bio-Rad Laboratories) according to the manufacturer’s protocol for serum samples and utilizing the recommended sample dilutions and standard curve concentrations. Acquisitions were performed using a Bio-Plex^®^ Manager v6.2 and Bio-Plex^®^ Data Pro™ Software v1.3 (Bio-Rad Laboratories). Values outside calibration curves were considered to be below the limit of detection.

### Statistical analysis

In this study, the cytokine storms were comprehensively defined by not only the expression levels but also CV. Cytokine concentrations at baseline (admission date or treatment start date; day 1) and at the end of the observation (patient outcome or last treatment date) were analyzed by the Steel-Dwass test for multiple comparisons. For comparisons of cytokine expression levels and CV between two or more independent groups, a mixed-effects regression model was used. Fixed effects included patient age, gender, disease severity (severe, moderate, mild, and no infection), and outcome (decease, transfer, discharge, and no infection). In addition, if the model did not include any infection samples, the observation period from admission and the patient as a random effect were included. Mean values and ranges (min–max) of clinical characteristics measured during the observation period for each subject were calculated. Differences between the groups concerning clinical characteristics were assessed using Fisher’s exact test or analysis of variance (ANOVA). Statistical analyses described above were performed using R v4.0.3 (R Foundation for Statistical Computing, Vienna, Austria) statistics software. A mixed-effects regression model analysis was executed with the R package lme4 and lmerTest. A *p*-value of < 0.05 (0.00035 when adjusted for Bonferroni correction) was considered statistically significant. The study was an exploratory data analysis with unknown effect sizes and confidence intervals for the hypotheses to be tested, and no statistical sample size calculations were performed. However, *post-hoc* powers with a mean of the cytokines were calculated with a sample size of 13 patients per group using a two-group *t*-test with a two-sided significance level of *p* < 0.05 to detect mean differences in comparing cytokine expression levels between COVID-19 patients and healthy volunteers.

## Results

### Patient classification in the COVID-19 cohort

In this study, a total of 134 serum samples were obtained from 23 adult COVID-19 patients treated at the University Hospital Kyoto Prefectural University of Medicine, Kyoto, Japan, during the third and fourth waves of COVID-19 that occurred in Japan from November 2020 to June 2021, and a total of 26 serum samples were obtained from 13 healthy volunteer subjects (Supplementary Figure S1A), in order to measure the level of cytokines circulating in the blood. One hundred and nine kinds of blood-circulating cytokines (Supplementary Table S1) were investigated using a fluorescent-labeled microbeads assay system. As the blood-circulating cytokine levels were measured over time from the identical patients, statistical analyses were performed using a mixed-effects regression model. Moreover, the cytokine levels were independently measured via multiple panels in a repeat method. The quantitative polymerase chain reaction (qPCR) method for SARS-CoV-2 viruses in nasopharyngeal swabs was used to determine COVID-19 positives at the Faculty of Clinical Laboratory. Twenty-three COVID-19 patients were selected randomly and classified into four severity groups based on clinical characteristics and the official Japanese Ministry of Health, Labour, and Welfare guideline for the management of COVID-19 (Supplementary Figure S1A). The main criteria were percutaneous oxygen saturation (SpO_2_) and intensive care unit (ICU) requirements. Some patients required oxygen therapy, which included non-mechanical and mechanical ventilation (MV) with oxygen. The subgroup of severe COVID-19 patients belonged to the requirement of ventilator management therapy [i.e., MV and extracorporeal membrane oxygenation (ECMO)] (*n* = 4), and the moderate II COVID-19 patients were included in the subgroup of SpO_2_ ≤ 93%, i.e., respiratory failure and the requirement of supplemental oxygen (*n* = 16). Moderate I COVID-19 patients were included in the subgroup of 93% < SpO_2_ < 96% and respiratory distress; however, those patients were not included in our cohort (*n* = 0). The mild COVID-19 patients had SpO_2_ ≥ 96% and no respiratory symptoms (*n* = 3). The clinical characteristics of the 23 patients are summarized in Supplementary Tables S2, S3. In brief, almost all COVID-19 patients examined had hypertension, diabetes mellitus, or chronic kidney disease, and a few patients had lung disease, malignant lymphoma, Parkinson’s disease, ulcerative colitis, ischemic heart disease, or immune-mediated thrombocytopenia (Supplementary Table S2). Patients were mainly treated using remdesivir (antiviral agent), dexamethasone (corticosteroid), prednisolone (corticosteroid), and tocilizumab (recombinant humanized anti-IL-6 receptor monoclonal antibody), along with oxygen administration (Supplementary Table S2). Moreover, the patients were also classified by hospitalization periods termed as “long-term” (≥5 weeks) and “short-term” (<5 weeks) (Supplementary Figure S1B). Patients with remission of COVID-19 symptoms were discharged without any additional therapies. Those who were no longer in severe condition but needed rehabilitation were transferred to affiliated hospitals. The University Hospital Kyoto Prefectural University of Medicine serves as a special functioning hospital that is authorized as a tertiary care facility for patients requiring intensive treatment including those with severe infectious diseases. The numbers of patients with decease, transfer, and discharge were 10, 7, and 6, respectively (Supplementary Figure S1B, Supplementary Table S2). The numbers of patients who stayed at the hospital in the long term and short term were 5 and 18, respectively (Supplementary Figure S1B, Supplementary Table S2). The timing of sampling during hospitalization covered over 78% of hospitalization periods in each patient (Supplementary Figure S2).

### Overview of the blood-circulating cytokine levels in COVID-19

**Figure 1:**
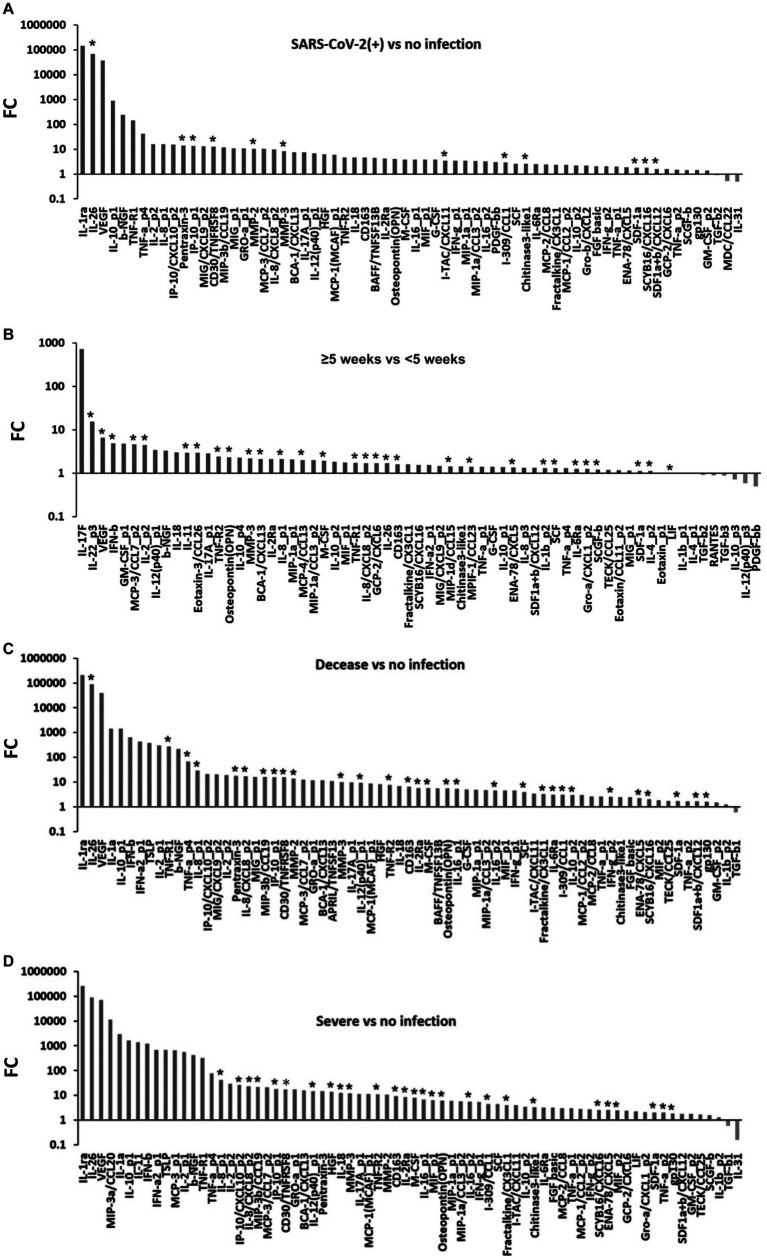
Differential expression of blood cytokines in COVID-19. **(A)** Markedly expressed cytokines in the COVID-19 patients compared to the healthy volunteer subjects (*p* < 0.05; mixed-effects regression model). **(B)** Markedly expressed cytokines in the long-term inpatients (≥5 weeks) compared to the short-term inpatients (<5 weeks) (*p* < 0.05; mixed-effects regression model). **(C)** Markedly expressed cytokines in the decease subgroup compared to the no infection subgroup (*p* < 0.05; mixed-effects regression model). **(D)** Markedly expressed cytokines in the severe subgroup compared to the no infection subgroup (*p* < 0.05; mixed-effects regression model). Asterisk (*): Bonferroni correction (*p* = 0.00035); FC, fold change.

The blood-circulating cytokine levels in 23 COVID-19 patients and 13 healthy volunteers were summarized for clinical outcomes, severity, and hospitalization period (Supplementary Table S3). In the patients analyzed, the gender ratio (male to female) was 2.28 (16 men to 7 women). The median age was 73 years (range: 20–91 years), and the duration of hospitalization was 20 days (range: 5–96 days). Similarly, at hospital admission before therapies, blood tests were conducted as follows: white blood cell (WBC) (×10^9^/L): 6.3 (1.1–18.1), hemoglobin (g/dL): 13.1 (8.7–16.4), platelet (×10^9^/L): 184.5 (20.0–401.0), D-dimer (μg/mL): 1.4 (0.6–20.3), lactate dehydrogenase (LD) (U/L): 437.0 (115.0–838.0), ferritin (ng/mL): 584.5 (106.0–10,565.0), and C-reactive protein (CRP) (mg/dL): 8.2 (0.1–31.2) (Supplementary Table S3). In comparison to the no infection subgroup, the expression levels of cytokines, especially IL-26 (median [interquartile range: IQR]: 585.44 [382.12–975.80] pg/mL, 68,136.81-fold), pentraxin-3 (47,892.44 [31,370.65–71,891.42] pg/mL, 13.90-fold), IP-10 (4,080.35 [2,458.71–6,672.04] pg/mL, 13.47-fold), sCD30 (2,686.48 [2,103.98–3,990.13] pg/mL, 12.65-fold), MMP-2 (31,544.07 [24,793.58–36,341.59] pg/mL, 10.40-fold), MMP-3 (27,590.05 [21,809.63–39,108.24] pg/mL, 8.27-fold), I-TAC (131.20 [82.31–166.80] pg/mL, 3.44-fold), I-309 (40.18 [28.89–57.50] pg/mL, 2.90-fold), CHI3L1 (26,700.92 [20,655.66–35,549.19] pg/mL, 2.57-fold), SDF1α + β (2,162.61 [1,915.03–2,602.42] pg/mL, 1.57-fold), and SCYB16 (739.40 [554.93–902.37] pg/mL, 1.78-fold), in the SARS-CoV-2 infection subgroup were extremely increased with significant differences with Bonferroni correction (*p* < 0.00035) (Figure 1A). On the other hand, IL-31 (25.24 [0.00–71.01] pg/mL, 0.49-fold), macrophage-derived chemokine (MDC) (258.61 [167.72–366.29] pg/mL, 0.52-fold), and TGF-β2 (3,850.26 [3,500.03–3,995.26] pg/mL, 0.91-fold) were decreased (*p* < 0.05) (Figure 1A). In the hospitalization period, the expression levels of cytokines, especially IL-22 (0.00 [0.00–0.00] pg/mL, 15.47-fold), IL-2 (41.09 [33.87–47.31] pg/mL, 4.48-fold), IL-11 (1.92 [0.00–9.82] pg/mL, 2.95-fold), IL-8 (200.62 [99.14–280.05] pg/mL, 1.69-fold), IL-26 (973.62 [749.68–1,311.64] pg/mL, 1.68-fold), IL-1β (6.57 [6.45–6.69] pg/mL, 1.31-fold), IL-4 (70.06 [56.63–76.18] pg/mL, 1.13-fold), VEGF (1,147.31 [1,143.48–1,622.59] pg/mL, 6.59-fold), IFN-β (3.09 [0.00–3.85] pg/mL, 4.92-fold), MCP-3 (106.99 [78.47–116.64] pg/mL, 4.63-fold), MCP-4 (148.30 [102.81–165.26] pg/mL, 2.01-fold), eotaxin-3 (225.44 [179.24–239.69] pg/mL, 2.95-fold), sTNF-R1 (7,762.24 [5,025.83–17,588.57] pg/mL, 1.72-fold), sTNF-R2 (2,746.18 [2,086.44–4,806.09] pg/mL, 2.42-fold), osteopontin (90,049.42 [70,454.42–104,808.06] pg/mL, 2.30-fold), MMP-3 (71,280.18 [51,496.25–74,096.90] pg/mL, 2.20-fold), BCA-1 (304.41 [264.00–452.77] pg/mL, 2.13-fold), M-CSF (76.79 [68.71–132.11] pg/mL, 1.91-fold), GCP-2 (72.90 [69.78–90.20] pg/mL, 1.69-fold), sCD163 (282,092.83 [257,135.99–700,110.78] pg/mL, 1.61-fold), MIP-1δ (7,852.14 [4,897.99–8,226.48] pg/mL, 1.44-fold), MPIF-1 (509.22 [348.72–680.76] pg/mL, 1.42-fold), ENA-78 (1,193.73 [1,147.85–1,305.69] pg/mL, 1.36-fold), stem cell factor (SCF) (197.40 [108.90–230.02] pg/mL, 1.31-fold), sIL-6Rα (25,743.16 [16,380.50–35,781.82] pg/mL, 1.27-fold), GRO-α (394.61 [278.31–471.72] pg/mL, 1.26-fold), SCGF-β (260,226.07 [252,813.31–291,356.01] pg/mL, 1.22-fold), SDF-1α (1,953.00 [1,842.71–2,159.84] pg/mL, 1.13-fold), and leukemia inhibitory factor (LIF) (41.30 [30.42–47.31] pg/mL, 1.01-fold), in the long-term hospitalization subgroup were extremely increased compared to the short-term hospitalization subgroup (*p* < 0.00035) (Figure 1B). Inversely, platelet-derived growth factor bb (PDGF-ββ) (1,749.67 [1,697.58–3,086.65] pg/mL, 0.50-fold), IL-12 (4.04 [0.00–5.01] pg/mL, 0.59-fold), IL-10 (0.00 [0.00–7.43] pg/mL, 0.72-fold), TGF-β3 (1,136.97 [1,123.09–1,372.05] pg/mL, 0.89-fold), TGF-β2 (3,850.26 [3,604.44–3,869.15] pg/mL, 0.92-fold), and RANTES (6,797.84 [4,044.30–8,535.95] pg/mL, 0.90-fold) were decreased (*p* < 0.05) (Figure 1B). These results suggested that various blood-circulating cytokines dramatically increase depending on the SARS-CoV-2 infection and severities requiring long-term hospitalization. On the other hand, a few cytokines were decreased with the SARS-CoV-2 infection. A sample size of 13 subjects per group corresponded to a mean *post-hoc* power of 0.81 (min = 0.30, max = 1.00) with 66 differentially expressed cytokines (*p* < 0.05).

### Differential expression of cytokines corresponding to severe clinical outcomes and disease severity

**Figure 2:**
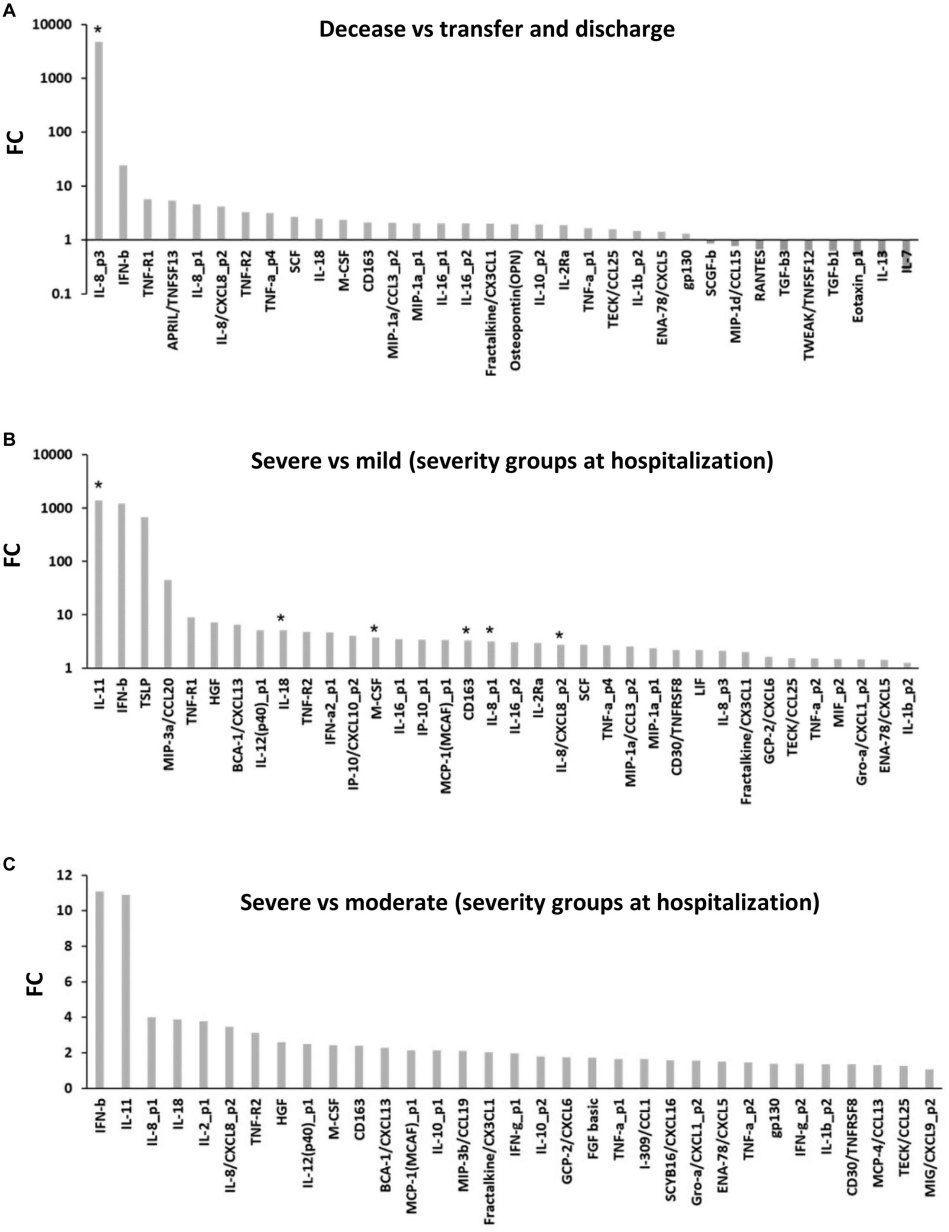
Clinical outcomes and disease severity-associated cytokines in COVID-19. **(A)** Markedly expressed cytokines in the decease subgroup compared to the transfer and discharge subgroups (*p* < 0.05; mixed-effects regression model). **(B,C)** Markedly expressed cytokines in the severe subgroup compared to the mild **(B)** and moderate **(C)** subgroups (*p* < 0.05; mixed-effects regression model). Asterisk (*): Bonferroni correction (*p* = 0.00035); FC, fold change.

Next, we attempted to determine the differential expression of cytokines corresponding to severe clinical outcomes and disease severity. In the decease subgroup compared to the no infection subgroup, 69 cytokines were markedly increased (*p* < 0.05) (Figure 1C). In specific, IL-26 (median [IQR]: 861.65 [458.98–1,278.02] pg/mL, 86,618.85-fold), IL-8 (114.64 [54.77–140.04] pg/mL, 28.74-fold), IL-12 (385.98 [210.04–523.26] pg/mL, 9.14-fold), IL-16 (159.41 [116.18–186.03] pg/mL, 5.31-fold), IL-10 (15.18 [9.10–18.72] pg/mL, 3.01-fold), sTNF-R1 (12,675.41 [5,079.70–26,175.81] pg/mL, 270.73-fold), sTNF-R2 (3,446.63 [1,867.74–4,703.10] pg/mL, 7.66-fold), TNF-α (12.09 [5.79–20.32] pg/mL, 67.85-fold), pentraxin-3 (59,299.30 [45,314.30–85,274.09] pg/mL, 17.64-fold), MIP-3β (182.16 [141.30–336.80] pg/mL, 16.04-fold), IP-10 (5,780.81 [3,033.90–6,917.04] pg/mL, 15.40-fold), sCD30 (3,605.39 [2,586.51–4,927.79] pg/mL, 15.31-fold), MMP-2 (33,740.70 [31,639.53–42,870.48] pg/mL, 13.79-fold), MMP-3 (38,073.69 [24,767.77–48,922.08] pg/mL, 9.97-fold), sCD163 (407,035.54 [253,100.61–769,089.47] pg/mL, 6.52-fold), sIL-2Rα (179.06 [131.66–290.17] pg/mL, 5.72-fold), M-CSF (93.10 [50.87–125.80] pg/mL, 5.63-fold), osteopontin (64,084.37 [49,198.38–88,504.00] pg/mL, 5.54-fold), SCF (213.71 [120.40–487.21] pg/mL, 3.98-fold), fractalkine (409.96 [328.90–627.89] pg/mL, 3.23-fold), sIL-6Rα (23,505.90 [17,595.14–35,248.49] pg/mL, 3.05-fold), I-309 (45.47 [22.75–60.26] pg/mL, 3.05-fold), IFN-γ (53.14 [43.10–68.50] pg/mL, 2.49-fold), ENA-78 (1,180.54 [1,030.94–1,336.41] pg/mL, 2.19-fold), SCYB-16 (862.16 [632.75–1,054.36] pg/mL, 2.01-fold), SDF1α + β (2,359.06 [2,088.08–2,624.00] pg/mL, 1.65-fold), and gp130 (135,605.43 [117,422.69–150,998.83] pg/mL, 1.62-fold) were extremely increased (*p* < 0.00035) (Figure 1C). However, TGF-β1 was decreased (*p* < 0.05) (Figure 1C). Of those, 37 cytokines were specific in the decease subgroup (Figure 1D). In the severe subgroup compared to the no infection subgroup, 72 cytokines were markedly increased (*p* < 0.05) (Figure 1D). In specific, IL-8 (143.50 [121.90–169.74] pg/mL, 40.98-fold), IL-12 (515.95 [488.11–696.09] pg/mL, 14.53-fold), IL-18 (363.91 [236.47–477.30] pg/mL, 12.29-fold), IL-16 (188.74 [167.98–217.29] pg/mL, 6.73-fold), IP-10 (3,120.36 [1,539.07–4,719.25] pg/mL, 25.85-fold), sCD163 (4,540.07 [3,917.65–4,839.25] pg/mL, 17.22-fold), MIP-3β (276.71 [199.99–392.59] pg/mL, 21.42-fold), hepatocyte growth factor (HGF) (3,063.01 [2,222.22–4,205.46] pg/mL, 13.58-fold), MMP-3 (49,435.11 [25,816.77–72,980.78] pg/mL, 12.04-fold), sTNF-R2 (4,792.95 [4,106.47–6,117.41] pg/mL, 11.10-fold), sCD163 (746,096.57 [595,606.29–901,475.49] pg/mL, 9.19-fold), sIL-2Rα (320.66 [265.01–415.75] pg/mL, 8.55-fold), M-CSF (131.03 [114.65–143.55] pg/mL, 7.77-fold), MIF (633.05 [593.25–759.77] pg/mL, 6.31-fold), osteopontin (80,251.92 [64,375.58–93,739.08] pg/mL, 5.99-fold), I-309 (59.24 [54.88–64.71] pg/mL, 4.42-fold), fractalkine (607.18 [522.45–688.25] pg/mL, 4.00-fold), CHI3L1 (35,549.19 [33,328.48–42,175.44] pg/mL, 3.33-fold), SCYB16 (1,041.27 [988.34–1,105.83] pg/mL, 2.55-fold), ENA-78 (1,326.17 [1,277.70–1,347.73] pg/mL, 2.50-fold), SDF-1α (1,988.95 [1,925.42–2,058.63] pg/mL, 2.01-fold), TNF-α (203.60 [200.59–223.28] pg/mL, 2.00-fold), and gp130 (149,444.36 [141,600.13–164,800.49] pg/mL, 1.83-fold) were extremely increased (*p* < 0.00035) (Figure 1D). However, IL-31 (23.39 [9.26–35.09] pg/mL, 0.16-fold) and TGF-β1 (39,668.11 [36,377.93–40,574.39] pg/mL, 0.60-fold) were decreased (*p* < 0.05) (Figure 1D). In the decease subgroup compared to the transfer or discharge subgroups, 38 cytokines were markedly increased (*p* < 0.05) (Figure 2A). In specific, IL-8 (50.37 [8.47–80.51] pg/mL, 4,674.04-fold) was extremely increased (*p* < 0.00035) (Figure 2A). However, IL-7 (0.35 [0.00–3.47] pg/mL, 0.25-fold), IL-13 (0.73 [0.42–1.17] pg/mL, 0.48-fold), eotaxin-1 (52.40 [44.72–61.67] pg/mL, 0.50-fold), TGF-β1 (39,668.11 [23,518.12–49,453.23] pg/mL, 0.63-fold), TGF-β3 (912.31 [797.22–1,133.50] pg/mL, 0.64-fold), TNF-like weak inducer of apoptosis (TWEAK) (267.17 [198.81–290.95] pg/mL, 0.64-fold), RANTES (5,470.80 [3,779.77–6,605.33] pg/mL, 0.66-fold), MIP-1δ (3,950.52 [2,894.01–4,961.29] pg/mL, 0.77-fold), and SCGF-β (237,082.22 [174,123.05–253,307.90] pg/mL, 0.86-fold) were decreased (*p* < 0.05) (Figure 2A). In the severe subgroup compared to the mild or moderate subgroups, 39 cytokines were markedly increased (*p* < 0.05) (Figures 2B,C). In specific, IL-11 (15.90 [7.37–22.45] pg/mL, 1,392.88-fold), IL-18 (363.91 [236.47–477.30] pg/mL, 5.10-fold), IL-8 (143.50 [121.90–169.74] pg/mL, 3.18-fold), M-CSF (131.03 [114.65–143.55] pg/mL, 3.76-fold), and sCD163 (746,096.57 [595,606.29–901,475.49] pg/mL, 3.27-fold) were extremely increased (*p* < 0.00035) (Figure 2B). No cytokines were decreased (Figures 2B,C). These findings suggest that approximately the same cytokines (i.e., IL-8, IL-12, M-CSF, sCD163, IP-10, MIP-3β, MMP-3, sTNF-R2, osteopontin, I-309, fractalkine, SCYB16, ENA-78, SDF-1, TNF-α, and gp130) seem to be increased in cases of severe clinical outcomes and disease severity (Supplementary Table S2). On the other hand, TGF-β1 would be decreased in the severe clinical outcomes and disease severity (Supplementary Table S2) in COVID-19.

### Temporal changes in cytokine levels associated with disease progression and recovery

**Figure 3:**
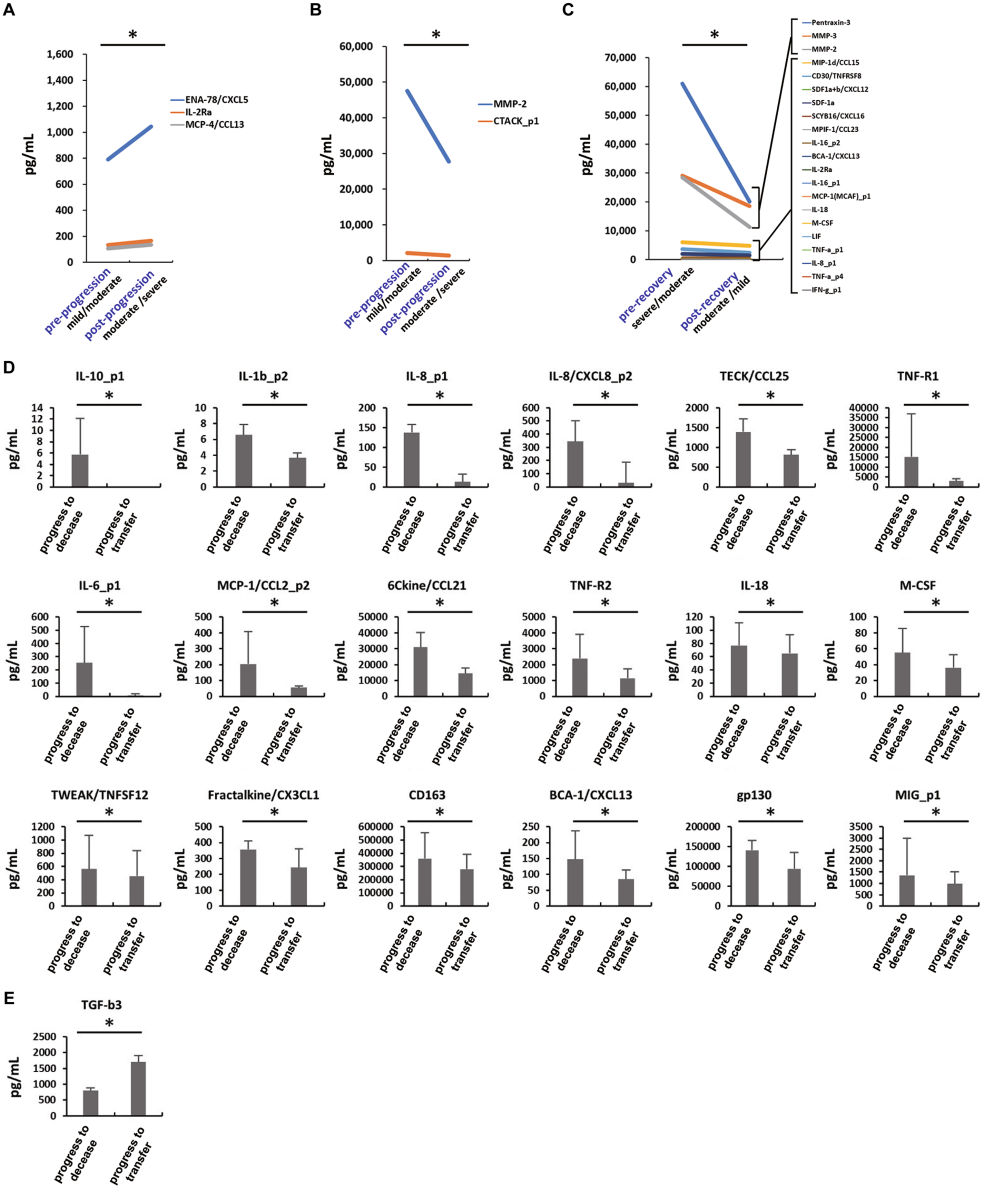
Local temporal expression changes of cytokine levels at severity and recovery. **(A,B)** Increase **(A)** and decrease **(B)** in cytokine levels at severity from mild and moderate (*n* = 2) to moderate and severe (*n* = 5), respectively. *p < 0.05: Wilcoxon signed-rank test. **(C)** Decreased cytokine levels at recovery from severe and moderate (*n* = 5) to moderate and mild (*n* = 2), respectively. *p < 0.05: Wilcoxon signed-rank test. **(D,E)** Last sampled blood cytokine levels indicating clinical outcomes as progress to decease (*n* = 4) and transfer (*n* = 5) from moderate and mild disease statuses at hospital admission. **(D)** Decreased are IL-10, IL-1β, IL-8, IL-6, IL-18, TECK, sTNF-R1, sTNF-R2, MCP-1, 6Ckine, M-CSF, TWEAK, fractalkine, sCD163, BCA-1, gp130, and MIG in the transfer compared to the decease subgroups. **(E)** TGF-β3 is increased in the transfer compared to the decease subgroups. *p < 0.05; Wilcoxon rank sum test.

Our findings on the local changes in cytokine expression levels during disease progression and recovery revealed that the expression of ENA-78 (mean: 790.76 to 1,043.67 pg/mL, 1.31-fold), MCP-4 (107.70 to 135.34 pg/mL, 1.26-fold), and sIL-2Rα (133.68 to 166.83 pg/mL, 1.25-fold) were temporally increased after disease progression in individual patients (i.e., mild to moderate; *n* = 2 or moderate to severe; *n* = 5) (*p* < 0.05) (Figure 3A), while cutaneous MMP-2 (47,558.94 to 27,729.73 pg/mL, 0.58-fold) and T-cell-attracting chemokine (CTACK) (2,123.97 to 1,445.90 pg/mL, 0.68-fold) were decreased in individual patients (*p* < 0.05) (Figure 3B). Temporal expression of pentraxin-3 (60,978.98 to 20,128.98 pg/mL, 0.33-fold), MMP-3 (29,093.80 to 18,580.21 pg/mL, 0.64-fold), and MMP-2 (28,463.28 to 11,250.30 pg/mL, 0.40-fold) were extremely decreased after disease recovery in individual patients (i.e., severe to moderate; *n* = 5 or moderate to mild; *n* = 2) (*p* < 0.05), while no cytokines were increased in individual patients (Figure 3C). Similarly, MIP-1δ, CD30, SDF-1, SCYB16, MPIF-1, IL-16, BCA-1, sIL-2Rα, MCP-1, IL-18, M-CSF, LIF, TNF-α, IL-8, and IFN-γ were also decreased after disease recovery in individual patients (*p* < 0.05) (Figure 3C). The increases of IL-10 (mean: 5.74 pg/mL in decease and 0.00 pg/mL in transfer), IL-1β (6.57 pg/mL and 3.66 pg/mL), IL-8 (345.34 pg/mL and 31.10 pg/mL), IL-6 (253.24 pg/mL and 6.08 pg/mL), IL-18 (76.83 pg/mL and 65.22 pg/mL), TECK (1,388.24 pg/mL and 814.26 pg/mL), sTNF-R1 (15,218.47 pg/mL and 3,072.05 pg/mL), sTNF-R2 (2,394.11 pg/mL and 1,142.10 pg/mL), MCP-1 (204.10 pg/mL and 54.87 pg/mL), 6Ckine (30,989.29 pg/mL and 14,480.94 pg/mL), M-CSF (55.19 pg/mL and 36.15 pg/mL), TWEAK (561.13 pg/mL and 452.24 pg/mL), fractalkine (357.11 pg/mL and 243.08 pg/mL), sCD163 (358,425.69 pg/mL and 279,630.70 pg/mL), BCA-1 (147.23 pg/mL and 84.78 pg/mL), gp130 (140,337.38 pg/mL and 94,292.02 pg/mL), and MIG (1,360.73 pg/mL and 988.76 pg/mL) (Figure 3D) or the decrease in TGF-β3 (802.56 pg/mL and 1,703.23 pg/mL) at the last sampling (Figure 3E) in hospital stay were observed in the patients who were deceased even if they were diagnosed moderate or mild severities at hospitalization (*n* = 4) (*p* < 0.05). Therefore, these findings suggest a possibility that the decreased levels of sIL-2Rα, ENA-78, IL-8, IL-18, MCP-1, MCP-4, M-CSF, and BCA-1 and/or the increased levels of TGF-β3 and CTACK, which might be required for recovery and survival from COVID-19. Whether such immunomodulators simply returned to normal range or represent primary processes responsible for clinical outcomes needs further investigation in the future. Based on the findings described above, we especially selected 11 cytokines that would be involved in the inflammation pathway in COVID-19, i.e., SDF-1, SCYB16, sCD30, IL-11, IL-18, IL-8, IFN-γ, TNF-α, sTNF-R2, M-CSF, and I-309 (Supplementary Figure S3). A sample size of 13 subjects per group returned a mean *post-hoc* power of 0.83 (min = 0.29, max = 1.00) with the 11 cytokines described above in comparing COVID-19 patients and healthy volunteers.

### Cytokine storm marker candidates in consideration of the CV of cytokine levels during the entire hospitalization period in COVID-19

**Figure 4:**
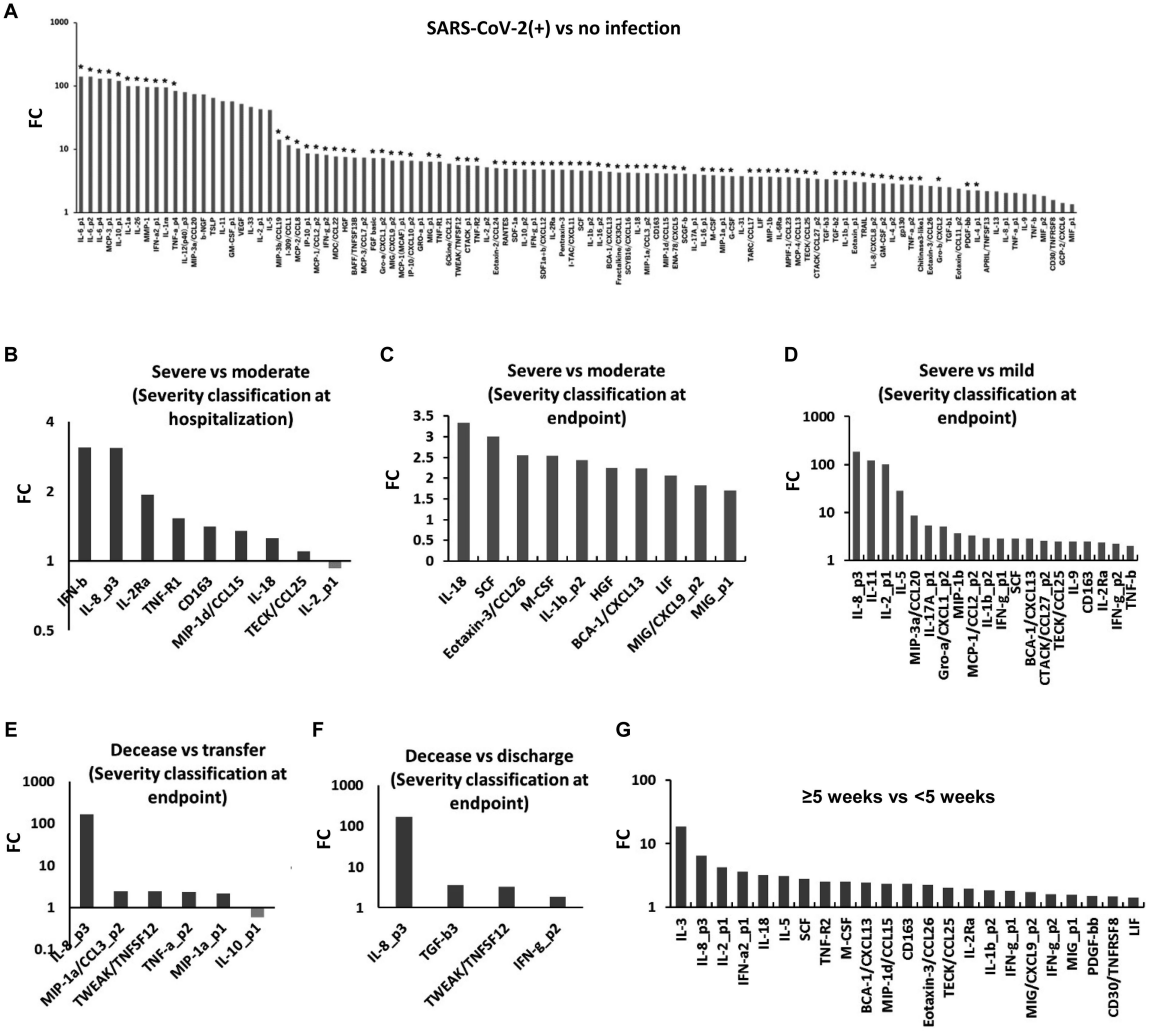
Alteration of coefficient of variation of blood cytokine levels in COVID-19. **(A)** Marked increase in the coefficient of variation (CV) in the COVID-19 patients compared to healthy volunteer subjects (*p* < 0.05; Wilcoxon rank sum test). **(B)** Increased CV in the severe subgroup compared to the moderate subgroup (*p* < 0.05; Steel-Dwass test for multiple comparisons). Disease severity was determined at hospitalization. **(C,D)** Increased CV of cytokines in the severe subgroup compared to the moderate **(C)** and mild **(D)** subgroups (*p* < 0.05; Steel-Dwass test for multiple comparisons). Disease severity was determined at the final measurement. **(E,F)** Increased CV in the severe subgroup compared to the transfer **(E)** and discharge **(F)** subgroups (*p* < 0.05; Steel-Dwass test for multiple comparisons). **(G)** Increased CV in the long-term (≥5 weeks) in patients compared to the short-term (<5 weeks) in patients (*p* < 0.05; Wilcoxon rank sum test). FC, fold change.

We also analyzed the CV of the cytokine levels during the entire hospitalization period of the patients. The CVs of these cytokines, especially IL-6 (median [IQR]: 1.35 [0.96–1.85], 140.95-fold), IL-10 (1.15 [0.85–1.68], 120.37-fold), IL-1α (1.08 [0.31–1.46], 99.89-fold), IL-26 (0.94 [0.38–1.37], 99.48-fold), MCP-3 (1.38 [0.66–1.87], 130.28-fold), MMP-1 (0.73 [0.21–1.30], 95.75-fold), IFN-α2 (0.69 [0.00–1.59], 95.61-fold), IL-1ra (0.87 [0.57–1.20], 94.89-fold), and TNF-α (0.78 [0.51–0.96], 83.64-fold), in the SARS-CoV-2 subgroup were extremely increased compared to the no infection subgroup, with significant differences with Bonferroni correction (*p* < 0.00035) (Figure 4A). Compared to the moderate and mild subgroups, the CVs of IFN-β, IFN-γ, IL-8, IL-18, IL-1β, IL-11, IL-5, IL-17, IL-9, sIL-2Rα, sTNF-R1, sCD163, MIP-1δ, MIP-3α, MIP-1β, TECK, SCF, eotaxin-3, M-CSF, HGF, BCA-1, LIF, MIG, GRO-α, MCP-1, CTACK, and TNF-β were increased in the severe subgroup (*p* < 0.05) (Figures 4B–D). Compared to the transfer and discharge subgroups, the CVs of IL-8, IL-10, MIP-1α, TWEAK, TNF-α, TGF-β3, and IFN-γ were increased in the decease group (*p* < 0.05) (Figures 4E,F). The CVs of IL-3, IL-8, IL-2, IL-18, IL-5, IL-1β, IFN-α2, IFN-γ, SCF, sTNF-R2, M-CSF, BCA-1, MIP-1δ, sCD163, sCD30, eotaxin-3, TECK, sIL-2Rα, MIG, PDGF-ββ, and LIF in the long-term hospitalization subgroup were increased compared to the short-term hospitalization subgroup (*p* < 0.05) (Figure 4G). These findings suggest a possibility that various blood-circulating cytokines, especially IL-10, IL-8, IL-18, IL-1β, IFN-α2, IFN-γ, TNF-α, sIL-2Rα, sCD163, MIP-1δ, TECK, SCF, eotaxin-3, M-CSF, BCA-1, LIF, MIG, sTNF-R1, and sTNF-R2, would be dysregulated in COVID-19.

## Discussion

In this study, matrix metalloproteinase MMP-3 and microenvironment remodeling factors including MCP-3, MIF, IL-8, SDF-1, and SCYB16 were detected as highly expressed cytokines in COVID-19. These findings suggested that potential treatment for COVID-19 should not only focus on conventional therapies targeting the immune pathway but also consider stabilizing and controlling microenvironment remodeling as a potential strategy. Here, we detected 76 cytokine marker candidates with Bonferroni-corrected significant differences in comparison with disease severity, clinical outcome, long-term hospitalization, and disease progression and recovery in COVID-19 (Appendix 1). On the other hand, this study also detected decreases of 19 cytokines (Appendix 2). Reduced cytokines might be also therapeutic targets for oxidative stress-related MAPK and JAK/STAT pathways, TGF-β signaling, and extracellular matrix (ECM) remodeling in COVID-19. These provide a hint for targeting therapy and anti-cytokines in COVID-19, but further studies are needed to confirm their efficacy in the future.

Previous COVID-19 studies have clarified several diagnostic markers and biomarkers, such as IP-10, CRP, and various ILs and IFNs (26) such as CD163 (27), MIF (28), IL-8 (29), IL-18 (30), FGF-basic (30), and CHI3L1 (31). Of great interest are MMP-3 as a progression marker (32), MCP-3 as a urine marker (33), and IL-2 as a heart disease marker (34). FGF-basic (35), CHI3L1 (36), and MCP-3 (37, 38) have also been identified as COVID-19 biomarkers with both transcriptome and proteome. Reportedly, SDF-1 recruits CD34+ hematopoietic stem/progenitor cells (39) and CD3-stimulated T-lymphocytes (40) into the virus-infected area. In addition, SDF-1 has fundamental roles in hematopoietic disruption, regeneration, and healing (39), which is the reason why plerixafor, also called Mozobil, the SDF-1 receptor antagonist has been used to protect CD34+ hematopoietic stem/progenitor cells. Therefore, SDF-1 may be involved in a wide range of COVID-19 symptoms and after-effects. Similarly, SCYB16 reportedly sequesters differentiated CD4+ T cells and natural killer T (NKT) cells around virus-infected cells (41). IL-11 plays a role in hematopoietic stem cell (HSC) differentiation into progenitor cells (42), which modulates and stabilizes reciprocal differentiation of CD4 + CD8+ cells into CD8+ NKT-cells and CD4+ helper T-cells via IL-11 signaling coupled with gp130 and their downstream JAK/STAT and Ras/MAPK signaling pathways for cell proliferation (42). Otherwise, IL-11 represses T-cell differentiation by activating CD11b + and CD14+ cells (43). CD4+ cells are differentiated into Th1 and Th2 by IL-12, IL-18, IL-27, and IFN-γ, and Th2 by IL-4, respectively (44). Although few studies have reported IL-11 in COVID-19, IL-11 might be a novel cytokine marker candidate in COVID-19. sCD30 binds CD30L (also known as CD153) or competes CD30-binding to CD30L on the cell surface (45, 46). The ratio of sCD30 and CD30 binding to CD30L could determine the Th1/Th2 balance, which would activate Th1 properties by IL-2, IFN-γ, and TNF and the JAK/STAT, MAPK, NF-κB, and sTNF-R2 signaling pathways, and also suppress the Th2 activities for B-cell class switching and antibody production, thus resulting in excessive cytokine release and hyperinflammation (47). Moreover, M-CSF differentiates HSCs into macrophages and other types of cells and plays roles in hematopoietic-lineage cell proliferation and differentiation (48). In addition, M-CSF activates macrophages and monocytes in their phagocytic and chemotactic activities (49). IL-18 is an integral membrane protein in M-CSF-differentiated macrophages with lipopolysaccharide stimulation and induces IFN-γ release from NK cells in a caspase-1-dependent fashion (50). IL-8 is also known to be a neutrophil chemotactic factor that induces the chemotaxis of neutrophils and granulocytes toward the virus infection area (51). IL-8 induces a series of physiological responses such as intracellular Ca^2+^ accumulation, exocytosis of substrate, and respiratory burst and is required for migration and phagocytosis of neutrophils and macrophages (52). I-309 binds to CCR8 on the cell surfaces of Th2 and Treg cells and activates these cells, competing with the hyperinflammation pathway via Th1 (53, 54). I-309-CCR8 signaling could modulate the Th1/Th2 balance determining disease progression and recovery. A recent study also demonstrates that M-CSF and I-309 markedly increased in the patients who ultimately died of COVID-19 (18). Thus, we propose a hypothetical model of the mechanism for the COVID-19 cytokine storm, in which SDF-1, SCYB16, IL-11, and sCD30, followed by M-CSF, IL-8, IL-12, IL-18, IFN-γ, sTNF-R2, and I-309 (Supplementary Figure S3), might play a pivotal role in hematopoietic stem/progenitor and helper T-cell differentiation and excessive cytokine release with hyperinflammation, yet further studies are needed to validate the proposal.

On the other hand, this study has also several issues as below. The unbalanced timing and distribution of sampling may cause a selection bias. The follow-up and information regarding transferred patients are missing; there is no information for these patients who survived or recovered. Considering the low number of patients in the study cohort, this may affect all the analyses performed and the differential expression of cytokines reported. *Post-hoc* statistical power is calculated for the COVID-19 marker subset constituted of the 11 cytokines (Supplementary Table S4). Cytokines with *post-hoc* statistical power > 0.7 are sCD30, SDF-1α, IFN-γ, SCYB16, M-CSF, I-309, TNF-α, IL-8, IL-18, and sTNF-R2 in SARS-CoV-2 infection vs. non-infection. Similarly, M-CSF, IL-8, IL-18, sTNF-R2, and IL-11 are detected by *post-hoc* statistical power > 0.7 in severe vs. moderate disease. In addition, the detected cytokines with *post-hoc* statistical power > 0.7 are sCD30 in severe vs. mild disease, IL-8 in decease vs. transfer, IFN-γ and IL-8 in decease vs. discharge, and IFN-γ, I-309, TNF-α, and IL-18 in long hospitalization (≥5 weeks) vs. short hospitalization (<5 weeks). However, due to the small sample size, the results need to be validated in a large cohort. In addition, this study mentions no demographic information or comorbidities of the patients and healthy controls, which may affect the cytokine levels. It is also important to use inclusion and exclusion criteria in this study, as many clinical parameters, such as secondary infections, intubation, mechanical ventilation, and thrombotic complications, may affect cytokine levels. Blood culture found that three patients were infected by bacteria. The one case was negative on reanalysis after days, and it is considered that there are few clinical effects. The other two cases were positive in the last specimens during follow-up, and it could not exclude a possibility of bacterial infection during a COVID-19 treatment (e.g., intubation) and hospital stay. In addition, four cases of ARDS were observed, all of which had hypoxemia (SpO_2_ < 90%), no cardiomegaly, and abnormal opacities in both lungs on computed tomography (CT) or X-ray imaging, according to ARDS Clinical Practice Guidelines 2016 and Berlin protocol. Therefore, in the four patients, it is considered that ARDS occurred following COVID-19 pneumonia. In general, although elevated levels of cytokines are known in COVID-19 patients with cytokine storm, these levels are much lower than in patients with ARDS or sepsis. This suggests that the COVID-19 cytokine storm is hard to evaluate because of the high expression of cytokines. Additionally, of the COVID-19 marker subset consisting of the 11 cytokines, there is no difference between the COVID-19 patients with diabetes comorbidity (*n* = 15) and those without it (*n* = 8) (Supplementary Table S5). However, at discharge and transfer, IL-18 in the patients with hemoglobin A1c (HbA1c) ≥7.0 is 1.96-fold higher than those with HbA1c <7.0 (*p* = 0.012) (Supplementary Table S6). In addition, I-309 is increased in the patients with a comorbidity of inflammatory bowel disease (*n* = 1) (Supplementary Figure S4). Furthermore, SDF-1α, SCYB16, and I-309 are also increased in the patients with thrombocytopenia (*n* = 1) (Supplementary Figure S4). Therefore, it must be carefully observed whether COVID-19 patients with high cytokine levels are associated with ARDS, sepsis, or comorbidities in a large cohort.

Previous studies have reported that various cytokines, interleukins, and chemokines including IL-6, IL-10, TNF-α, and IFN-γ are stimulated with COVID-19 infection (3, 15–18, 20). Although many of the cytokines detected in the study have already been reported as possible diagnostic marker candidates (3–6, 16, 18, 20), IL-11 might also be a novel diagnostic marker candidate in COVID-19. These findings could develop personalized medicine with recombinant proteins and anti-chemokine drugs, e.g., the recombinant IL-11, oprelvekin, to stimulate the proliferation of HSCs followed by the anti-SDF-1 reagent, plerixafor, to mobilize HSCs around infected cells. On the other hand, reactivation of the signaling pathways involved in the decreased cytokines, such as IL-31, chemokines RANTES, MDC, PDGF, and TGF-β family members, might also be a novel strategy for COVID-19 therapy. While there is extensive research into biomarkers, studies on biological relevance might be relatively understudied in the context of COVID-19 around the world. Our findings also suggested that the cytokines increased with COVID-19 infection, compared to no infection, yet decreased by mortality, severe disease, and progression. Further detailed analyses in large populations are required for investigations of potential confounding factors, confirmations of the claims and proposals of this study, and generalizability of these findings to different populations.

In summary, we here described the cytokine storm precisely by investigating not only the expression level but also the range of fluctuations during hospitalization as CV, which would be a novel insight for evaluating the COVID-19 cytokine storm. Based on our findings, we considered that ECM remodeling might be a therapeutic target in addition to the conventional anti-interleukin treatment targeting the immune and inflammatory pathways. In addition, we proposed a hypothetical model that SDF-1, SCYB16, IL-11, sCD30, and I-309 might all play a pivotal role in helper T-cell differentiation and excessive cytokine release with immune response and inflammation in COVID-19.

## Data availability statement

The original contributions presented in the study are included in the article/Supplementary materials, further inquiries can be directed to the corresponding authors.

## Ethics statement

The studies involving humans were approved by the Institutional Review Board at Kyoto Prefectural University of Medicine (ERB-G-109 and ERB-C-1810). The studies were conducted in accordance with the local legislation and institutional requirements. Written informed consent for participation in this study was provided by the participants' legal guardians/next of kin.

## Author contributions

YaT: Conceptualization, Data curation, Formal analysis, Investigation, Project administration, Resources, Supervision, Validation, Writing – original draft, Writing – review & editing. TI: Conceptualization, Data curation, Formal analysis, Investigation, Resources, Supervision, Validation, Writing – original draft, Writing – review & editing. TM: Investigation, Resources, Writing – original draft, Writing – review & editing. KY: Data curation, Formal analysis, Investigation, Methodology, Resources, Validation, Visualization, Writing – original draft, Writing – review & editing. MT: Data curation, Formal analysis, Investigation, Resources, Validation, Writing – original draft, Writing – review & editing. KM: Investigation, Resources, Writing – original draft, Writing – review & editing. KS: Investigation, Resources, Writing – original draft, Writing – review & editing. YuT: Investigation, Resources, Writing – original draft, Writing – review & editing. NO: Investigation, Resources, Writing – original draft, Writing – review & editing. MN: Investigation, Resources, Writing – original draft, Writing – review & editing. TN: Investigation, Resources, Writing – original draft, Writing – review & editing. NF: Investigation, Resources, Writing – original draft, Writing – review & editing. CS: Investigation, Resources, Writing – original draft, Writing – review & editing. TS: Conceptualization, Data curation, Formal analysis, Funding acquisition, Project administration, Supervision, Validation, Writing – original draft, Writing – review & editing. KT: Formal analysis, Funding acquisition, Project administration, Supervision, Validation, Writing – original draft, Writing – review & editing, Conceptualization, Data curation. BO: Conceptualization, Data curation, Formal analysis, Funding acquisition, Project administration, Supervision, Validation, Writing – original draft, Writing – review & editing.
