# Supplementary material for: Potential marker subset of blood-circulating cytokines on hematopoietic progenitor-to-Th1 pathway in COVID-19
Source: Front Med (Lausanne). 2024 Feb 27;11:1319980. doi: 10.3389/fmed.2024.1319980 (PMC10927758; doi:10.3389/fmed.2024.1319980)
Supplement: Supplementary file 1 [file Data_Sheet_1.pdf]

Potential marker subset of blood circulating cytokines on hematopoietic progenitor-to-Th1 pathway in COVID-19

Yasuo Takashima[1,†], Tohru Inaba[2,†], Tasuku Matsuyama[3,†], Kengo Yoshii[4], Masami Tanaka[1], Kazumichi Matsumoto[5], Kazuki Sudo[6], Yuichi Tokuda[1], Natsue Omi[1], Masakazu Nakano[1], Takaaki Nakaya[7], Naohisa Fujita[2,8], Chie Sotozono[9], Teiji Sawa[6,*], Kei Tashiro[1,*] & Bon Ohta[3,*]

[1] Department of Genomic Medical Sciences, Kyoto Prefectural University of Medicine, Kyoto, Japan.
[2] Department of Infection Control and Laboratory Medicine, Kyoto Prefectural University of Medicine, Kyoto, Japan.
[3] Department of Emergency Medicine, Kyoto Prefectural University of Medicine, Kyoto, Japan.
[4] Department of Mathematics and Statistics in Medical Sciences, Kyoto Prefectural University of Medicine, Kyoto, Japan.
[5] Faculty of Clinical Laboratory, University Hospital Kyoto Prefectural University of Medicine, Kyoto, Japan.
[6] Department of Anesthesiology, Kyoto Prefectural University of Medicine, Kyoto, Japan.
[7] Department of Infectious Diseases, Kyoto Prefectural University of Medicine, Kyoto, Japan.
[8] Kyoto Prefectural Institute of Public Health and Environment, Kyoto, Japan.
[9] Department of Ophthalmology, Kyoto Prefectural University of Medicine, Kyoto, Japan.

† These authors contributed equally to this work.

*** Correspondence and Address for Reprints:**

Teiji Sawa, M.D., Ph.D., Department of Anesthesiology, Kyoto Prefectural University of Medicine, 465 Kajii-cho, Hirokoji-agaru, Kawaramachi-dori, Kamigyo-ku, Kyoto 602-8566, Japan. Tel: +81-75-251-5633, Fax: +81-75-251-5843, E-mail: anesth@koto.kpu-m.ac.jp

Kei Tashiro, M.D., Ph.D., Department of Genomic Medical Sciences, Kyoto Prefectural University of Medicine, 465 Kajii-cho, Hirokoji-agaru, Kawaramachi-dori, Kamigyo-ku, Kyoto 602-8566, Japan. Tel: +81-75-251-5346, Fax: +81-75-251-5347, E-mail: tashiro@koto.kpu-m.ac.jp

Bon Ohta, M.D., Ph.D., Department of Emergency Medicine, Kyoto Prefectural University of Medicine, 465 Kajii-cho, Hirokoji-agaru, Kawaramachi-dori, Kamigyo-ku, Kyoto 602-8566, Japan. Tel: +81-75-251-5393, Fax: +81-75-251-5393, E-mail: b-ohta@koto.kpu-m.ac.jp

Supplementary Information

Appendixes

APPENDIX 1 | The candidates of the COVID-19 cytokine storm components associated with the infection, disease severity, long-term hospitalization, severe clinical outcome, and disease progression and recovery. BAFF (TNFSF13B), BCA-1 (CXCL13), sCD163, sCD30 (TNFRSF8), Chitinase3-like1 (CHI3L1), CTACK (CCL27), ENA-78 (CXCL5), Eotaxin, Eotaxin-2 (CCL24), Eotaxin-3 (CCL26), FGF-basic, Fractalkine (CX3CL1), GCP-2 (CXCL6), G-CSF, GM-CSF, gp130, Gro-α (CXCL1), Gro-β (CXCL2), HGF, I-309 (CCL1), IFN-α2, IFN-β, IFN-γ, IL-10, IL-11, IL-12 (p40), IL-16, IL-18, IL-1α, IL-1β, IL-2, IL-22, IL-26, IL-4, IL-6, IL-8 (CXCL8), IL-1ra (receptor antagonist), sIL-2Rα (receptor alpha), sIL-6Rα (receptor alpha), IP-10 (CXCL10), I-TAC (CXCL11), LIF, MCP-1 (CCL2), MCP-2 (CCL8), MCP-3 (CCL7), MCP-4 (CCL13), M-CSF, MDC (CCL22), MIF, MIG (CXCL9), MIP-1α (CCL3), MIP-1β, MIP-1δ (CCL15), MIP-3β (CCL19), MMP-1, MMP-2, MMP-3, MPIF-1 (CCL23), Osteopontin (OPN), PDGF-ββ, Pentraxin-3, RANTES, SCF, SCGF-β, SCYB16 (CXCL16), SDF-1α (CXCL12), TARC (CCL17), TECK (CCL25), TGF-β2, TNF-α, sTNF-R1, sTNF-R2, TRAIL, TWEAK (TNFSF12), VEGF.

Here we detected 76 cytokine marker candidates with Bonferroni-corrected significant differences in comparison with disease severity, clinical outcome, long-term hospitalization, and disease progression and recovery in COVID-19 (**Appendix 1**). Of these, IP-10, sTNF-R1, sTNF-R2, sCD30, sCD163, HGF, SCYB16, IL-16, MIG, SDF-1, and FKN are considered major components of the COVID-19 cytokine storm. The characteristics of COVID-19 diagnostic biomarker candidates were clarified as follows; (i) IL-6, IL-10, IL-1α, IL-26, MCP-3, MMP-1, IFN-α2, IL-1ra, and TNF-α for SARS-CoV-2 infection, (ii) IL-22, IL-2, IL-3, IL-8, VEGF, IFN-β, and MCP-3 for long-term hospitalization, (iii) IL-1ra, IL-26, IL-11, IL-18, IL-8, IL-2, VEGF, IFN-β, and SCF for outcomes of severe and lethal, and (iv) sIL-2Rα, MCP-4, MCP-1, ENA-78, MMP-2, MMP-3, CTAK, LIF, SCYB16, IFN-β, MIP-1δ, MPIF, SDF-1, M-CSF, IL-18, IL-8, IL-16, IL-10, IL-1β, IL-6, sCD30, BCA-1, TNF-α, pentraxin-3, TECK, sTNF-R1, and TGF-β3 for the local temporal expression changes in disease progression and recovery. Consequently, we selected 11 cytokines, including SDF-1, SCYB16, sCD30, IL-11, IL-18, IL-8, IFN-γ, TNF-α, sTNF-R2, M-CSF, and I-309, as a potential cytokine marker subset for infection, mortality, disease severities including progression, recovery, and long hospitalization.

APPENDIX 2 | The reduced cytokines according to COVID-19 infection, severe symptoms, disease progression and mortality. CTACK (CCL27), Eotaxin (CCL11), IL-10, IL-12, IL-13, IL-1β, IL-31, IL-4, IL-7, MDC (CCL22), MIP-1δ (CCL15), MMP-2, PDGF-ββ (PDGF2), RANTES (CCL5), SCGF-β (CLEC11A), TGF-β1, TGF-β2, TGF-β3, TWEAK (TNFSF12).

The study also detected the decreases of 19 cytokines (**Appendix 2**). In specific, TGF-β1 (0.52-fold), MDC (0.56-fold), TGF-β3 (0.57-fold), thymus and activation-regulated chemokine (TARC) (0.67-fold), eotaxin (0.67-fold), IL-

9 (0.82-fold), and TNF-β (0.91-fold) in the Decease subgroup were decreased at endpoint compared with the averages in the No-infection subgroup samples. Moreover, lower levels of TARC, PDGF-ββ, TWEAK, TGF-β3, TGF-β1, SCGF-β, and eotaxin were detected in 50% of the deceased patients compared with the healthy controls.

List of Supplementary Tables

SUPPLEMENTARY TABLE 1 | Cytokines examined in the study.

SUPPLEMENTARY TABLE 2 | Clinical information of COVID-19 patients. Note: OA; oxygen administration, MV; mechanical ventilation, ECMO; extracorporeal membrane oxygenation, HD; hemodialysis. HT; hypertension, DM; diabetes mellitus, CKD; chronic renal disease, IHD; ischemic heart disease, ITP; immune-mediated thrombocytopenia. #1: These patients needed the mechanical ventilation at the time of transfer, but subsequently became free from ventilator. #2: This patient needed oxygen inhalation at the time of transfer provably due to the underlying lung disease (interstitial pneumonia).

SUPPLEMENTARY TABLE 3 | Clinical characteristics of COVID-19 patients. Note: Numbers were presented by median (min - max). †, Kruskal-Wallis test. ‡, Fisher's exact test. NA, not applicable.

SUPPLEMENTARY TABLE 4 | Post-hoc power analysis for 11 cytokine storm markers in COVID-19. NOTE: Bold represents post-hoc power >0.7.

SUPPLEMENTARY TABLE 5 | Difference of cytokines of interest in diabetes mellitus in comorbidity with COVID-19. Note: DM; diabetes mellitus, *p-values with Wilcoxon rank sum test. Sorted by p-value.

SUPPLEMENTARY TABLE 6 | Comparative analysis of cytokine levels by HbA1c groups in COVID-19 patients. Note: DM; diabetes mellitus, HbA1c; hemoglobin A1c. Sorted by p-values with Wilcoxon rank sum test.

Supplementary Figure Legends

SUPPLEMENTARY FIGURE 1 | Criteria used in the study. Flow chart to classify disease severity and clinical outcome. **(A)** Flow chart to classify disease severity. ECMO: extracorporeal membrane oxygenation; ICU: intensive care unit; MV: mechanical ventilation; SpO_2: percutaneous oxygen saturation. **(B)** The numbers of patients diagnosed at hospitalization. Clinical outcomes were classified into decease, transfer, and discharge. Long and short hospitalization were divided by 5 weeks.

SUPPLEMENTARY FIGURE 2 | Timing of sampling during hospitalization and the follow up period after transfer and discharge. Horizontal lines indicate time-courses of hospitalization of patients and healthy volunteers. Vertical lines indicate sampling timing. Top numbers represent the day of sampling from hospital admission. The numbers

of samples and the follow-up periods are shown at the left of the horizontal bars.

SUPPLEMENTARY FIGURE 3 | A hypothetical model constituted of a potential marker subset of blood circulating cytokines in COVID-19. **(i)** CD34+ hematopoietic stem-progenitor cells are differentiated to CD3-stimulated T cells with M-CSF and IL-11 signaling, followed by differentiation to CD4+CD8+ cells. **(ii)** Stabilizing for CD8+ NKT and CD4+ helper T-cells by IL-11 signaling followed by JAK-STAT and Ras-MAPK signaling pathways or suppression of CD4+CD8+ differentiation via recruiting CD11b+ and CD14+ cells. IL-11 and M-CSF also play a role for differentiation to CD34+ progenitor cells from stem cells. CD4+ helper T-cells are differentiated into Th1 cells by IL-12, IL-18, and IFN-γ, and Th2 cells. **(iii)** Activation of Th1 and Th2 helper T-cells by sCD30 and I-309 binding to CD30L and CCR8 on the cell surfaces, respectively. **(iv)** Cell accumulation into virus-infected area of CD34+ hematopoietic stem/progenitor and CD3-stimulated T cells by M-CSF and SDF-1, and of NKT cells by SCYB16. **(v)** The Th1/Th2 balance determines disease progression and recovery in COVID-19. I-309 binds CCR8 on Th2 and Treg. Activated Th2 and Treg binding I-309 antagonize Th1 and suppress hyperinflammation. **(vi)** IFN-γ activates the JAK-STAT, MAPK, NF-κB, and TNF-R2 signaling pathways in Th1 cells, which then leads to the excessive release of cytokines and causes hyperinflammation.

SUPPLEMENTARY FIGURE 4 | Relative expression of blood circulating cytokines of interest in inflammatory bowel disease and thrombocytopenia in comorbidity with COVID-19 in the cohort. Disease severity was diagnosed at hospital stay admission. Expression levels of cytokines of interest are plotted in the COVID-19 patients. Rectangle; inflammatory bowel disease (IBD), triangle; thrombocytopenia, black circle; severe, gray circle; moderate, open circle; mild.

SUPPLEMENTARY TABLE 1 | Cytokines examined in the study.

symbol	name	cytokine	cytokine panel (p1)	inflammation panel (p2)	chemokine panel (p3)	TGF-β panel (p4)	Th-17 panel (p5)
BGLAP	Osteocalcin	Osteocalcin		Osteocalcin			
CCL1	Chemokine (C-C motif) ligand 1	I-309/CCL1			I-309/CCL1		
CCL11	Eotaxin/C-C motif chemokine 11	Eotaxin/CCL11	Eotaxin/CCL11		Eotaxin/CCL11		
CCL13	Monocyte chemoattractant protein-4	MCP-4/CCL13			MCP-4/CCL13		
CCL15	Macrophage inflammatory protein-1δ	MIP-1δ/CCL15			MIP-1δ/CCL15		
CCL17	Thymus and activation-regulated chemokine	TARC/CCL17			TARC/CCL17		
CCL19	Macrophage inflammatory protein-3β	MIP-3β/CCL19			MIP-3β/CCL19		
CCL2	Monocyte chemoattractant protein 1, monocyte chemotactic and activating factor	MCP-1(MCAF)/CCL2	MCP-1(MCAF)/CCL2		MCP-1(MCAF)/CCL2		
CCL20	Macrophage inflammatory protein-3α	MIP-3α/CCL20			MIP-3α/CCL20		
CCL21	Chemokine (C-C motif) ligand 21, six conserved cysteine residues	6Ckine/CCL21			6Ckine/CCL21		
CCL22	Dendritic cells and macrophages	MDC/CCL22			MDC/CCL22		
CCL23	Myeloid progenitor inhibitory factor 1	MPIF-1/CCL23			MPIF-1/CCL23		
CCL24	Eosinophil chemotactic protein 2	Eotaxin-2/CCL24/MPIF2			Eotaxin-2/CCL24/MPIF2		
CCL25	Thymus-Expressed Chemokine	TECK/CCL25			TECK/CCL25		
CCL26	Eosinophil chemotactic protein 3	Eotaxin-3/CCL26			Eotaxin-3/CCL26		
CCL27	cutaneous T-cell-attracting chemokine	CTACK/CCL27	CTACK/CCL27		CTACK/CCL27		
CCL3	Macrophage inflammatory protein-1α	MIP-1α/CCL3	MIP-1α/CCL3		MIP-1α/CCL3		
CCL4	Macrophage inflammatory protein-1β	MIP-1β	MIP-1β				
CCL5	regulated on activation, normal T cell expressed and secreted	RANTES	RANTES				
CCL7	Monocyte chemoattractant protein 3	MCP-3/CCL7	MCP-3/CCL7		MCP-3/CCL7		
CCL8	Monocyte chemoattractant protein-2	MCP-2/CCL8			MCP-2/CCL8		
CD163	Soluble CD163	sCD163		sCD163			
CD40LG	Soluble CD40 ligand	sCD40L					sCD40L
CHI3L1	Chitinase-3-like1	Chitinase-3-like1		Chitinase-3-like1			
CLEC11A	stem cell growth factor beta	SCGF-β	SCGF-β				
CSF1	Macrophage colony stimulating factor	M-CSF	M-CSF				
CSF2	Granulocyte macrophage colony-stimulating Factor	GM-CSF	GM-CSF		GM-CSF		
CSF3	Granulocyte colony stimulating factor	G-CSF	G-CSF				
CX3CL1	Fractalkine/chemokine (C-X3-C motif) ligand 1	Fractalkine/CX3CL1			Fractalkine/CX3CL1		
CXCL1	Growth-regulated protein alpha	Gro-α/CXCL1	Gro-α/CXCL1		Gro-α/CXCL1		
CXCL10	Interferon-γ inducible protein 10 kDa	IP-10/CXCL10	IP-10/CXCL10		IP-10/CXCL10		
CXCL11	Interferon-inducible T-cell alpha chemoattractant	I-TAC/CXCL11			I-TAC/CXCL11		
CXCL12	Stromal cell-derived factor-1α	SDF-1α	SDF-1α				
CXCL12	Stromal cell-derived factor 1	SDF-1α/CXCL12			SDF-1α/CXCL12		
CXCL13	B lymphocyte chemoattractant (BLC) or B cell-attracting chemokine 1/Chemokine (C-X-C motif) ligand 13	BCA-1/CXCL13			BCA-1/CXCL13		
CXCL16	Chemokine (C-X-C motif) ligand 16	SCYB16/CXCL16			SCYB16/CXCL16		
CXCL2	Growth-regulated protein beta	Gro-β/CXCL2			Gro-β/CXCL2		
CXCL5	Epithelial-derived neutrophil-activating peptide 78	ENA-78/CXCL5			ENA-78/CXCL5		
CXCL6	Granulocyte chemotactic protein 2	GCP-2/CXCL6			GCP-2/CXCL6		
CXCL8	Interleukin-8	IL-8/CXCL8	IL-8/CXCL8	IL-8/CXCL8	IL-8/CXCL8		
CXCL9	monokine induced by gamma interferon	MIG/CXCL9	MIG/CXCL9		MIG/CXCL9		
EBI3	Interleukin-35	IL-35		IL-35			
FGF2	Basic fibroblast growth factor	FGF-basic	FGFbasic				
HGF	Hepatocyte growth factor	HGF	HGF				
IFNA2	Interferon-α2	IFN-α2	IFN-α2	IFN-α2			
IFNB1	Interferon-β	IFN-β		IFN-β			
IFNG	Interferon-ϒ	IFN-ϒ	IFN-ϒ	IFN-ϒ	IFN-ϒ		IFN-ϒ
IFNL1	Interleukin-29	IL-29/IFN-λ1		IL-29/IFN-λ1			
IFNL2	Interleukin-28	IL-28A/IFN-λ2		IL-28A/IFN-λ2			
IL10	Interleukin-10	IL-10	IL-10	IL-10	IL-10		IL-10
IL11	Interleukin-11	IL-11		IL-11			
IL12A	Interleukin-12(p70)	IL-12(p70)	IL-12(p70)	IL-12(p70)			
IL12B	Interleukin-12(p40)	IL-12(p40)	IL-12(p40)	IL-12(p40)			
IL13	Interleukin-13	IL-13	IL-13				
IL15	Interleukin-15	IL-15	IL-15				
IL16	Interleukin-16	IL-16	IL-16		IL-16		
IL17F	Interleukin-17F	IL-17F					IL-17F
IL17RA	Interleukin-17A	IL-17/IL-17A	IL-17/IL-17A				IL-17/IL-17A
IL18	Interleukin-18	IL-18	IL-18				
IL19	Interleukin-19	IL-19		IL-19			
IL1A	Interleukin-1α	IL-1α	IL-1α				
IL1B	Interleukin-1β	IL-1β	IL-1β		IL-1β		IL-1β
IL1RN	Interleukin-1 receptor antagonist	IL-1ra	IL-1ra				
IL2	Interleukin-2	IL-2	IL-2	IL-2	IL-2		
IL20	Interleukin-20	IL-20		IL-20			
IL21	Interleukin-21	IL-21					IL-21
IL22	Interleukin-22	IL-22		IL-22			IL-22
IL23A	Interleukin-23	IL-23					IL-23
IL25	Interleukin-25	IL-25					IL-25
IL26	Interleukin-26	IL-26		IL-26			
IL27	Interleukin-27	IL-27(p28)		IL-27(p28)			
IL2RA	Interleukin-2 receptor α	IL-2Rα	IL-2Rα				
IL3	Interleukin-3	IL-3	IL-3				
IL31	Interleukin-31	IL-31					IL-31
IL32	Interleukin-32	IL-32		IL-32			
IL33	Interleukin-33	IL-33					IL-33
IL34	Interleukin-34	IL-34		IL-34			
IL4	Interleukin-4	IL-4	IL-4		IL-4		IL-4
IL5	Interleukin-5	IL-5	IL-5				
IL6	Interleukin-6	IL-6	IL-6		IL-6		IL-6
IL6R	Soluble Interleukin-6 receptor α	sIL-6Rα		sIL-6Rα			
IL6ST	gp130/soluble IL-6 receptor β	gp130/sIL-6Rβ		gp130/sIL-6Rβ			
IL7	Interleukin-7	IL-7	IL-7				
IL9	Interleukin-9	IL-9	IL-9				
KITLG	Stem cell factor	SCF	SCF				
LIF	Leukemia inhibitory factor	LIF	LIF				
LTA	Tumor necrosis factor-β	TNF-β	TNF-β				
MIF	Macrophage migration inhibitory factor	MIF	MIF		MIF		
MMP1	Matrix metalloproteinase-1	MMP-1		MMP-1			
MMP2	Matrix metalloproteinase-2	MMP-2		MMP-2			
MMP3	Matrix metalloproteinase-3	MMP-3		MMP-3			
NGF	Nerve growth factor β	β-NGF	β-NGF				
PDGFB	Platelet-derived growth factor-ββ	PDGF-ββ	PDGF-BB				
PTX3	Pentraxin 3	Pentraxin-3		Pentraxin-3			
SPP1	Osteopontin	Osteopontin		Osteopontin			
TGFB1	Transforming growth factor-β1	TGF-β1				TGF-β1	
TGFB2	Transforming growth factor-β2	TGF-β2				TGF-β2	
TGFB3	Transforming growth factor-β3	TGF-β3				TGF-β3	

TNF	Tumor necrosis factor-α	TNF-α	TNF-α		TNF-α	TNF-α
TNFRSF1A	Soluble TNF receptor-1	sTNF-R1		sTNF-R1		
TNFRSF1B	Soluble TNF receptor-2	sTNF-R2		sTNF-R2		
TNFRSF8	Soluble CD30	sCD30/TNFRSF8		sCD30/TNFRSF8		
TNFSF10	TNF-related apoptosis-inducing ligand	TRAIL	TRAIL			
TNFSF12	TNF-related weak inducer of apoptosis	TWEAK/TNFSF12		TWEAK/TNFSF12		
TNFSF13	a proliferation-inducing ligand)	APRIL/TNFSF13		APRIL/TNFSF13		
TNFSF13B	B-cell activating factor	BAFF/TNFSF13B		BAFF/TNFSF13B		
TNFSF14	homologous to lymphotoxin, exhibits inducible expression and competes with HSV glycoprotein D for binding to herpesvirus entry mediator, a receptor expressed on T lymphocytes	LIGHT/TNFSF14		LIGHT/TNFSF14		
TSLP	Thymic stromal lymphopoietin	TSLP		TSLP		
VEGFA	Vascular endothelial growth factor	VEGF	VEGF			

SUPPLEMENTARY TABLE 2 | Clinical information of COVID-19 patients.

gender	age	underlying illness	specific medication for COVID19	intensive care	days of hospitalization	severity on admission	severest phase	severity at last obsevation	outcome at last obsevation	cause of death
M	51	HT, DM	remdesivir, dexamethasone, prednisolone, tocilizumab	OA, MV, ECMO	96	severe	severe	severe	decease	respiratory failure
M	58	HT, DM, CKD	favipiravir, remdesivir, dexamethasone	OA, MV, ECMO, HD	74	severe	severe	severe	decease	respiratory failure
M	64	DM, CKD	remdesivir, dexamethasone, prednisolone	OA, MV, HD	52	severe	severe	severe	decease	respiratory failure
M	70	HT, DM, CKD, IHD	remdesivir, dexamethasone, prednisolone	OA, MV,	12	moderate II	severe	severe	decease	respiratory failure
M	73	DM, CKD	favipiravir, dexamethasone	OA, HD	24	moderate II	moderate II	moderate II	decease	acute myocardial infarction
M	73	DM, CKD, IHD	remdesivir, dexamethasone, prednisolone	MV	32	severe	severe	severe	decease	respiratory failure
M	79	DM, IHD, lung disease	favipiravir	OA	7	mild	moderate II	moderate II	decease	lung disease (lung cancer)
M	80	DM, malignant lymhoma	remdesivir, dexamethasone	OA, MV,	72	moderate II	severe	severe	decease	respiratory failure
F	85	HT, CKD	remdesivir, dexamethasone, tocilizumab	OA	21	moderate II	moderate II	moderate II	decease	respiratory failure
M	91	CKD	remdesivir, dexamethasone	OA	26	moderate II	moderate II	moderate II	decease	respiratory failure
F	43	-	favipiravir, dexamethasone	OA	5	moderate II	moderate II	mild	transfer	
F	67	Parkinson's disease	favipiravir, remdesivir, dexamethasone	OA,	7	moderate II	moderate II	mild	transfer	
M	77	HT, DM,	favipiravir, dexamethasone	OA, MV,	33	moderate II	severe	severe(#1	transfer	
F	79	ITP	favipiravir, dexamethasone	OA	20	moderate II	moderate II	moderate II	transfer	
F	81	HT, DM	remdesivir, dexamethasone, prednisolone, tocilizumab	OA, MV,	20	moderate II	severe	moderate I	transfer	
M	81	DM	remdesivir, dexamethasone	OA	13	moderate II	moderate II	moderate II	transfer	
M	81	DM, lung disease	remdesivir, dexamethasone, prednisolone, tocilizumab	OA, MV,	66	moderate II	severe	severe(#1	transfer	
M	20	ulcerative colitis	remdesivir, dexamethasone, prednisolone		32	mild	mild	mild	discharge	
M	66	HT, lung disease	remdesivir, dexamethasone, prednisolone	OA	16	moderate II	moderate II	mild	discharge	
M	70	DM, IHD	remdesivir, dexamethasone	OA	15	mild	moderate II	mild	discharge	
M	72	DM, lung disease	remdesivir, prednisolone	OA	17	moderate II	moderate II	moderate II (#2	discharge	
F	74	HT, DM	favipiravir, remdesivir, dexamethasone	OA	20	moderate II	moderate II	mild	discharge	
F	86	HT	remdesivir		18	mild	mild	mild	discharge	

Note: OA; oxygen administration, MV; mechanical ventilation, ECMO; extracorporeal membrane oxygenation, HD; hemodialysis. HT; hypertension, DM; diabetes mellitus, CKD; chronic renal disease, IHD; ischemic heart disease, ITP; immune-mediated thrombocytopenia. #1: These patients needed the mechanical ventilation at the time of transfer, but subesequently became free from ventilator. #2: This patient needed oxygen inhalation at the time of transfer provably due to the underlying lung disease (interstitial pneumonia)

SUPPLEMENTARY TABLE 3 | Clinical characteristics of COVID-19 patients.

	SARS-CoV-2(+):Total	SARS-CoV-2(+):Severe	SARS-CoV-2(+):Moderate II	SARS-CoV-2(+):Mild	Internal comparison (P value†)	Healthy volunteer
case number	23	4	15	4		13
male	16	4	9	3	0.537‡	10
female	7	0	6	1		3
age	73 (20 - 91)	61 (51 - 73)	78 (44 - 91)	75 (20 - 87)	0.119	62 (45–70)
hospitalization period	20 (5 - 96)	63 (32 - 96)	20 (5 - 72)	17 (7 - 32)	0.025	NA
**laboratory data**						
white blood cell (WBC) (x 10^9/L)	6.3 (1.1 - 18.1)	8.5 (2.9 - 11.0)	5.6 (1.1 - 18.1)	7.0 (4.2 - 7.8)	0.910	NA
Neut. (%)	83.3 (48.7 - 97.9)	86.3 (82.6 - 94.3)	83.2 (48.7 - 97.9)	78.3 (65.0 - 89.7)	0.573	NA
Lymph. (%)	9.7 (0.7 - 45.0)	8.3 (2.6 - 13.9)	10.0 (0.7 - 45.0)	14.5 (2.8 - 20.1)	0.714	NA
Mon. (%)	4.8 (1.3 - 18.5)	3.3 (3.0 - 7.6)	4.3 (1.3 - 18.5)	7.5 (6.9 - 13.5)	0.248	NA
Eosin. (%)	0.0 (0.0 - 0.9)	0.0 (0.0 - 0.0)	0.0 (0.0 - 0.2)	0.0 (0.0 - 0.9)	0.359	NA
Bas. (%)	0.1 (0.0 - 0.5)	0.1 (0.0 - 0.2)	0.1 (0.0 - 0.2)	0.3 (0.0 - 0.5)	0.403	NA
hemoglobin (g/dL)	13.1 (8.7 - 16.4)	13.3 (12.2 - 14.8)	13.3 (8.7 - 16.4)	10.6 (10.3 - 12.0)	0.143	NA
platelet (x 10^9/L)	184.5 (20.0 - 401.0)	100.5 (81.0 - 238.0)	184.0 (20.0 - 317.0)	319.0 (229.0 - 401.0)	0.061	NA
D-dimer (μg/mL)	1.4 (0.6 - 20.3)	8.0 (1.1 - 14.8)	1.9 (0.6 - 20.3)	1.2 (0.9 - 1.5)	0.596	NA
lactate dehydrogenase (LD) (U/L)	437.0 (115.0 - 838.0)	513.5 (429.0 - 838.0)	437.5 (267.0 - 700.0)	291.0 (115.0 - 370.0)	0.040	NA
asparatate aminoitransferase (AST) (U/L)	34.0 (14.0 - 211.0)	67.5 (51.0 - 211.0)	31.0 (14.0 - 108.0)	34.0 (16.0 - 34.0)	0.047	NA
alanine aminotransferase (ALT) (U/L)	19.0 (7.0 - 149.0)	48.5 (42.0 - 68.0)	17.5 (7.0 - 149.0)	19.0 (12.0 - 22.0)	0.026	NA
alkaline phosphatase (ALP) (U/L)	217.5 (141.0 - 370.0)	204.0 (176.0 - 350.0)	219.0 (147.0 - 370.0)	183.0 (141.0 - 259.0)	0.596	NA
γ-glutamyl transferase (γ-GT) (U/L)	34.0 (11.0 - 616.0)	109.0 (67.0 - 616.0)	28.5 (13.0 - 238.0)	27.0 (11.0 - 34.0)	0.027	NA
creatine phosphokinase (CK) (U/L)	127.0 (21.0 - 2365.0)	349.0 (141.0 - 2365.0)	75.0 (21.0 - 1899.0)	145.5 (133.0 - 158.0)	0.039	NA
HbA1c (%)	6.8 (5.4 - 8.9)	7.1 (7.0 - 7.1)	6.8 (5.5 - 8.9)	6.4 (5.4 - 7.6)	0.614	NA
total protein (g/dL)	6.2 (4.6 - 6.9)	6.0 (4.6 - 6.6)	6.1 (4.6 - 6.9)	6.2 (5.2 - 6.9)	0.883	NA
albumin (g/dL)	2.7 (1.9 - 3.5)	2.7 (2.2 - 3.2)	2.9 (1.9 - 3.5)	2.6 (2.5 - 2.9)	0.477	NA
total bilirubin (T-Bil.) (mg/dL)	0.6 (0.2 - 1.6)	0.8 (0.7 - 0.9)	0.5 (0.2 - 1.6)	0.6 (0.2 - 0.7)	0.201	NA
direct bilirubin (D-Bil.) (mg/dL)	0.5 (0.5 - 0.5)	0.5 (0.5 - 0.5)	NA	NA	NA	NA
urea nitrogen (UN) (mg/dL)	23.2 (1.7 - 108.0)	42.8 (37.4 - 44.6)	22.8 (1.7 - 108.0)	12.9 (9.8 - 13.0)	0.019	NA
creatinine (CRE) (mg/dL)	0.8 (0.4 - 10.6)	2.6 (0.8 - 10.6)	0.8 (0.6 - 8.3)	0.8 (0.4 - 1.1)	0.083	NA
ferritin (ng/mL)	584.5 (106.0 - 10565.0)	1126.0 (365.0 - 2768.0)	1113.0 (198.0 - 10565.0)	191.0 (106.0 - 203.0)	0.045	NA
C-reactive protein (CRP) (mg/dL)	8.2 (0.1 - 31.2)	10.0 (0.1 - 31.2)	10.3 (1.9 - 20.4)	4.5 (0.2 - 8.9)	0.523	NA
brain natriuretic peptide (BNP) (pg/mL)	430.1 (192.8 - 667.4)	192.8 (192.8 - 192.8)	667.4 (667.4 - 667.4)	NA	0.317	NA

Note: Numbers were presented by median (min - max). †, Kruskal-Wallis test. ‡, Fisher's exact test. NA, not applicable.

SUPPLEMENTARY TABLE 4 | Post-hoc power analysis for 11 cytokine storm markers in COVID-19.

Cytokine	SARS-CoV-2(+) vs. No infection	Severe vs. Moderate	Severe vs. Mild	Decease vs. Transfer	Decease vs. Discharge	Hospital stay ≥5w vs. <5w
sCD30	**1.00**	0.16	**0.73**	0.18	0.43	0.13
SDF-1α	**1.00**	0.08	0.12	0.13	0.19	0.11
IFN-γ	**0.97**	0.21	0.22	0.24	**0.76**	**0.90**
SCYB16	**0.97**	0.51	0.65	0.15	0.18	0.63
M-CSF	**0.91**	**0.80**	0.67	0.61	0.61	0.41
I-309	**0.87**	0.30	0.40	0.03	0.08	**0.79**
TNF-α	**0.85**	0.40	0.59	0.16	0.34	**0.91**
IL-8	**0.82**	**0.96**	0.45	**0.81**	**0.78**	0.26
IL-18	**0.72**	**0.98**	0.54	0.32	0.30	**0.73**
sTNF-R2	**0.71**	**0.71**	0.34	0.55	0.53	0.43
IL-11	0.30	**0.81**	0.33	0.18	0.11	0.15

NOTE: Bold represents post-hoc power >0.7.

SUPPLEMENTARY TABLE 5 | Difference of cytokines of interest in diabetes mellitus in comorbidity with COVID-19.

	DM (n=15)					non-DM (n=8)					
	min	25th percentile	median	75th percentile	max	min	25th percentile	median	75th percentile	max	*p**
IL-18	49.9	72.2	120.7	153.8	1174.6	41.0	58.4	71.2	89.2	151.2	0.065
SCYB16	382.9	611.0	778.9	881.9	1375.3	202.1	381.8	482.6	704.4	1237.7	0.101
M-CSF	26.5	36.7	54.7	95.5	256.9	28.6	31.1	34.3	49.5	74.2	0.149
CD30	1122.2	1831.4	2798.2	3897.9	4696.1	988.7	1865.8	2071.1	2696.6	3191.9	0.238
TNF-α	0.0	0.8	3.6	8.4	32.5	0.0	4.0	6.3	8.4	19.5	0.355
TNF-R2	732.0	1049.1	1202.2	2704.0	10756.0	739.3	905.7	1155.5	1426.3	2144.0	0.392
I-309	0.0	29.7	37.0	46.1	75.1	0.0	9.9	31.3	45.1	65.7	0.475
IL-11	0.0	0.0	0.0	0.0	9.8	0.0	0.0	0.0	0.0	0.0	0.526
IL-8	21.2	35.4	76.0	94.0	161.6	25.4	33.6	54.5	76.6	95.4	0.548
IFN-γ	15.1	40.0	43.9	51.5	114.2	29.0	38.5	49.2	51.0	72.5	0.825
SDF1$\alpha+\beta$	1387.2	1709.3	2142.2	2786.0	3935.8	1576.0	1729.9	2028.1	2929.8	4571.1	0.875
SDF-1α	797.1	1434.1	1803.1	2465.2	2962.3	880.8	1518.2	1743.0	2196.4	3700.7	0.925

Note: DM; diabetes mellitus, *p-values with Wilcoxon rank sum test. Sorted by p-value.

SUPPLEMENTARY TABLE 6 | Comparative analysis of cytokine levels by HbA1c groups in COVID-19 patients.

	HbA1c ≥7.0					HbA1c <7.0					
	min	25th percentile	median	75th percentile	max	min	25th percentile	median	75th percentile	max	p-value
IL-18	68.6	89.6	130.0	187.2	1174.6	42.8	55.6	66.0	89.2	151.2	0.012
M-CSF	31.5	37.2	72.6	96.2	256.9	26.5	31.5	37.4	54.4	74.2	0.101
IFN-g	35.6	41.9	46.6	53.0	114.2	15.1	33.3	41.4	48.2	49.9	0.173
SDF1a+b	1474.8	1572.4	1964.9	2278.5	3684.6	1387.2	2006.7	2566.4	3120.3	4571.1	0.203
SCYB16	470.2	620.7	732.4	877.7	1375.3	202.1	388.7	503.5	912.6	1237.7	0.203
IL-11	0.0	0.0	0.0	0.0	9.8	0.0	0.0	0.0	0.0	0.0	0.444
CD30	1122.2	1715.4	2797.5	3779.2	4696.1	988.7	1788.5	1966.8	2887.7	4349.8	0.515
IL-8	21.2	44.7	76.3	93.8	101.0	25.4	33.3	45.7	78.0	148.9	0.573
TNF-R2	732.0	1026.8	1246.9	1935.1	10756.0	739.3	969.1	1134.0	1770.6	2144.0	0.633
SDF-1a	797.1	1441.6	1638.6	2009.9	2962.3	880.8	1345.7	2005.1	2202.5	3700.7	0.897
TNF-a	0.0	2.0	4.3	6.9	14.6	0.0	0.9	4.4	8.0	19.5	0.911
I-309	0.0	30.2	34.2	39.6	66.0	0.0	3.3	36.5	61.3	75.1	0.980

Note: DM; diabetes mellitus, HbA1c; hemoglobin A1c. Sorted by p-values with Wilcoxon rank sum test.

SUPPLEMENTARY FIGURE 1

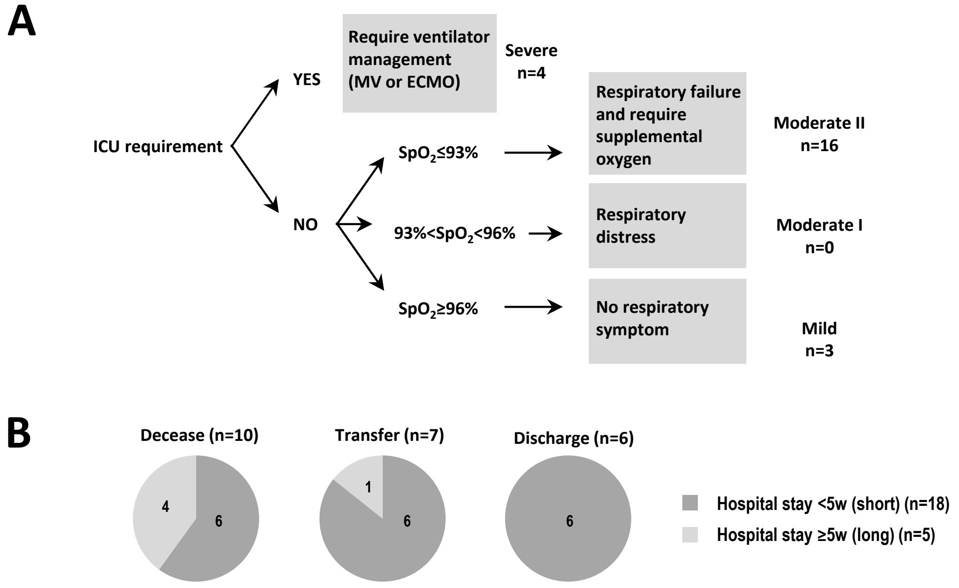

SUPPLEMENTARY FIGURE 1 | Criteria used in the study. Flow chart to classify disease severity and clinical outcome. **(A)** Flow chart to classify disease severity. ECMO: extracorporeal membrane oxygenation; ICU: intensive care unit; MV: mechanical ventilation; SpO_2: percutaneous oxygen saturation. **(B)** The numbers of patients diagnosed at hospitalization. Clinical outcomes were classified into decease, transfer, and discharge. Long and short hospitalization were divided by 5 weeks.

SUPPLEMENTARY FIGURE 2

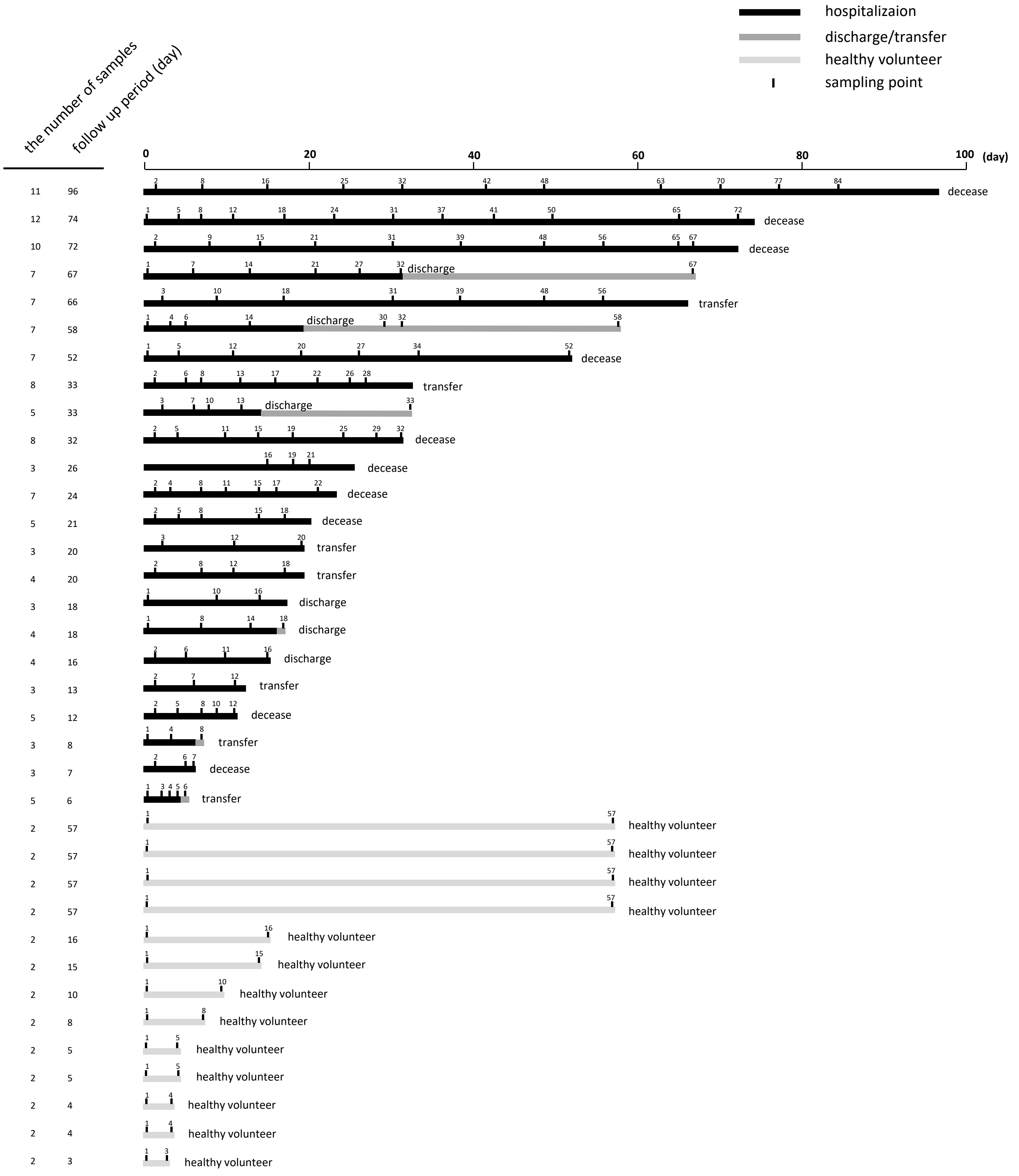

SUPPLEMENTARY FIGURE 2 | Timing of sampling during hospitalization and the follow up period after transfer and discharge. Horizontal lines indicate time-courses of hospitalization of patients and healthy volunteers. Vertical lines indicate sampling timing. Top numbers represent the day of sampling from hospital admission. The numbers of samples and the follow-up periods are shown at the left of the horizontal bars.

SUPPLEMENTARY FIGURE 3

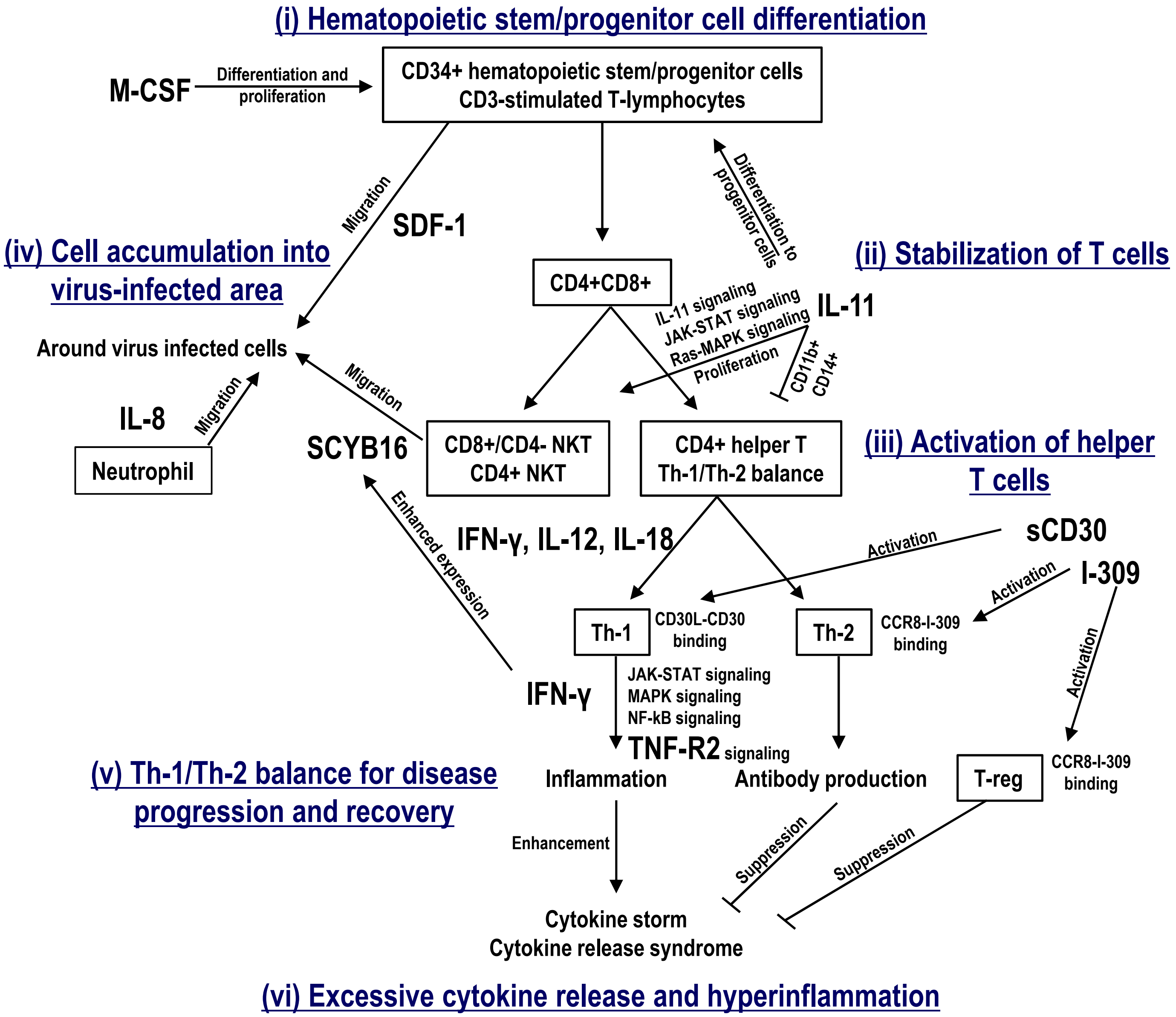

SUPPLEMENTARY FIGURE 3 | A hypothetical model constituted of a potential marker subset of blood circulating cytokines in COVID-19. **(i)** CD34+ hematopoietic stem-progenitor cells are differentiated to CD3-stimulated T cells with M-CSF and IL-11 signaling, followed by differentiation to CD4+CD8+ cells. **(ii)** Stabilizing for CD8+ NKT and CD4+ helper T-cells by IL-11 signaling followed by JAK-STAT and Ras-MAPK signaling pathways or suppression of CD4+CD8+ differentiation via recruiting CD11b+ and CD14+ cells. IL-11 and M-CSF also play a role for differentiation to CD34+ progenitor cells from stem cells. CD4+ helper T-cells are differentiated into Th1 cells by IL-12, IL-18, and IFN-γ, and Th2 cells. **(iii)** Activation of Th1 and Th2 helper T-cells by sCD30 and I-309 binding to CD30L and CCR8 on the cell surfaces, respectively. **(iv)** Cell accumulation into virus-infected area of CD34+ hematopoietic stem/progenitor and CD3-stimulated T cells by M-CSF and SDF-1, and of NKT cells by SCYB16. **(v)** The Th1/Th2 balance determines disease progression and recovery in COVID-19. I-309 binds CCR8 on Th2 and Treg. Activated Th2 and Treg binding I-309 antagonize Th1 and suppress hyperinflammation. **(vi)** IFN-γ activates the JAK-STAT, MAPK, NF-κB, and TNF-R2 signaling pathways in Th1 cells, which then leads to the excessive release of cytokines and causes hyperinflammation.

SUPPLEMENTARY FIGURE 4

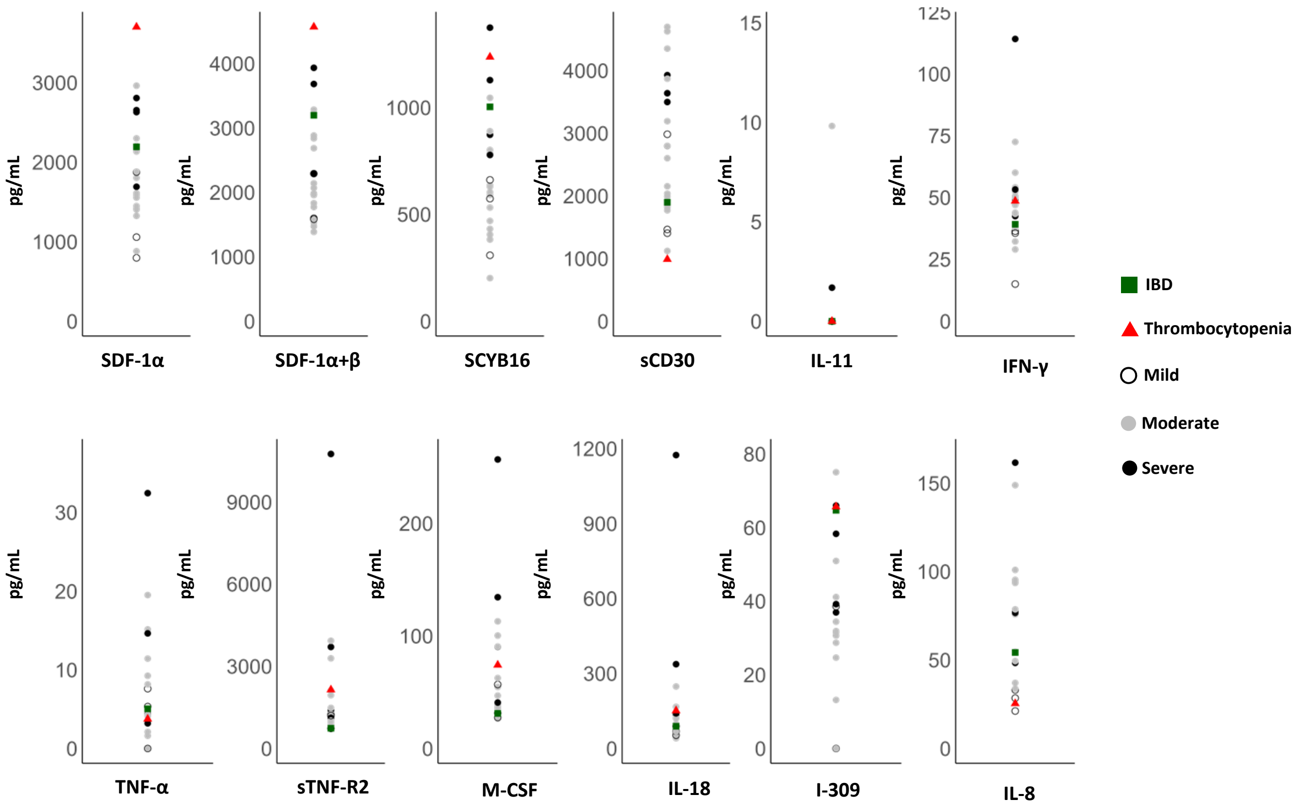

SUPPLEMENTARY FIGURE 4 | Relative expression of blood circulating cytokines of interest in inflammatory bowel disease and thrombocytopenia in comorbidity with COVID-19 in the cohort. Disease severity was diagnosed at hospital stay admission. Expression levels of cytokines of interest are plotted in the COVID-19 patients. Rectangle; inflammatory bowel disease (IBD), triangle; thrombocytopenia, black circle; severe, gray circle; moderate, open circle; mild.
